# The Zinc-Finger Antiviral Protein ZAP Inhibits LINE and Alu Retrotransposition

**DOI:** 10.1371/journal.pgen.1005121

**Published:** 2015-05-07

**Authors:** John B. Moldovan, John V. Moran

**Affiliations:** 1 Cellular and Molecular Biology Graduate Program, University of Michigan, Ann Arbor, Michigan, United States of America; 2 Departments of Human Genetics and Internal Medicine, University of Michigan, Ann Arbor, Michigan, United States of America; 3 Howard Hughes Medical Institute, University of Michigan, Ann Arbor, Michigan, United States of America; Fred Hutchinson Cancer Research Center, UNITED STATES

## Abstract

Long INterspersed Element-1 (LINE-1 or L1) is the only active autonomous retrotransposon in the human genome. To investigate the interplay between the L1 retrotransposition machinery and the host cell, we used co-immunoprecipitation in conjunction with liquid chromatography and tandem mass spectrometry to identify cellular proteins that interact with the L1 first open reading frame-encoded protein, ORF1p. We identified 39 ORF1p-interacting candidate proteins including the zinc-finger antiviral protein (ZAP or ZC3HAV1). Here we show that the interaction between ZAP and ORF1p requires RNA and that ZAP overexpression in HeLa cells inhibits the retrotransposition of engineered human L1 and Alu elements, an engineered mouse L1, and an engineered zebrafish LINE-2 element. Consistently, siRNA-mediated depletion of endogenous ZAP in HeLa cells led to a ~2-fold increase in human L1 retrotransposition. Fluorescence microscopy in cultured human cells demonstrated that ZAP co-localizes with L1 RNA, ORF1p, and stress granule associated proteins in cytoplasmic foci. Finally, molecular genetic and biochemical analyses indicate that ZAP reduces the accumulation of full-length L1 RNA and the L1-encoded proteins, yielding mechanistic insight about how ZAP may inhibit L1 retrotransposition. Together, these data suggest that ZAP inhibits the retrotransposition of LINE and Alu elements.

## Author Summary

Long INterspersed Element-1 (LINE-1 or L1) is the only active autonomous retrotransposon in the human genome. L1s comprise ~17% of human DNA and it is estimated that an average human genome has ~80–100 active L1s. L1 moves throughout the genome via a “copy-and-paste” mechanism known as retrotransposition. L1 retrotransposition is known to cause mutations; thus, it stands to reason that the host cell has evolved mechanisms to protect the cell from unabated retrotransposition. Here, we demonstrate that the zinc-finger antiviral protein (ZAP) inhibits the retrotransposition of human L1 and Alu retrotransposons, as well as related retrotransposons from mice and zebrafish. Biochemical and genetic data suggest that ZAP interacts with L1 RNA. Fluorescent microscopy demonstrates that ZAP associates with L1 in cytoplasmic foci that co-localize with stress granule proteins. Mechanistic analyses suggest that ZAP reduces the expression of full-length L1 RNA and the L1-encoded proteins, thereby providing mechanistic insight for how ZAP may restricts retrotransposition. Importantly, these data suggest that ZAP initially may have evolved to combat endogenous retrotransposons and subsequently was co-opted as a viral restriction factor.

## Introduction

Long INterspersed Element-1 (LINE-1, also known as L1) sequences comprise ~17% of human DNA and represent the only class of autonomously active retrotransposons in the genome [1]. L1s mobilize (*i*.*e*., retrotranspose) throughout the genome via an RNA intermediate by a copy-and-paste mechanism known as retrotransposition [reviewed in 2]. The overwhelming majority of human L1s are retrotransposition-deficient because they are 5' truncated, contain internal rearrangements (*i*.*e*., inversion/deletion events), or harbor point mutations that compromise the functions of the L1-encoded proteins (ORF1p and ORF2p) [1,3]. Despite these facts, it is estimated that the average diploid human genome contains ~80–100 L1 elements that are capable of retrotransposition [4–6]. It is estimated that a new L1 insertion occurs in approximately 1 out of 200 live human births [reviewed in 7]. On occasion, L1 retrotransposition events can disrupt gene expression, leading to diseases such as hemophilia A [8], Duchenne muscular dystrophy [9], and cancer [10,11]. Indeed, L1-mediated retrotransposition events are responsible for at least 96 disease-producing insertions in man [reviewed in 12].

A full-length human L1 is ~6 kb in length and encodes a 5' UTR that harbors an internal RNA polymerase II promoter that directs transcription from at or near the first base of the element [13–15]. The 5' UTR is followed by two open reading frames (ORFs) that are separated by a short 63 bp inter-ORF spacer, and a 3' UTR that ends in a variable length poly adenosine (poly(A)) tract [16,17]. The first L1 ORF encodes an ~40 kDa protein (ORF1p) that has nucleic acid binding [18–22] and nucleic acid chaperone activities [22,23]. The second L1 ORF encodes a much larger ~150 kDa protein (ORF2p) [24–26], which exhibits single-strand endonuclease (EN) [27] and reverse transcriptase (RT) [28,29] activities. Experiments in cultured cells have revealed that activities associated with both ORF1p and ORF2p are required for efficient L1 retrotransposition [27,30].

During a cycle of L1 retrotransposition, a full-length L1 is transcribed and the resultant bicistronic L1 mRNA is exported to the cytoplasm where it undergoes translation. Notably, L1 RNA is translated in a cap-dependent manner by an unconventional termination-reinitiation mechanism that facilitates translation of both L1 ORFs [31–34]. Following translation, ORF1p and ORF2p preferentially bind to their respective encoding L1 mRNA template (a phenomenon known as *cis*-preference [35,36]) to form an L1 ribonucleoprotein particle (RNP) [18,19,26,37,38]. Components of the L1 RNP gain access to the nucleus by a process that does not strictly require cell division [39], although L1 retrotransposition seems to be enhanced in dividing cells [40,41]. Once the L1 RNP has entered the nucleus, the L1 RNA is reverse transcribed and inserted into genomic DNA by a process known as target-site primed reverse transcription (TPRT) [27,42,43]. Briefly, the ORF2p endonuclease generates a single-strand endonucleolytic nick in genomic DNA at a thymidine rich consensus sequence (*e*.*g*., 5'-TTTT/A, 5'-TCTT/A, 5'-TTTA/A, *etc*.) [27,44,45]. The resulting 3' hydroxyl group then is used by the ORF2p reverse transcriptase as a primer to initiate (-) strand L1 cDNA synthesis from the L1 mRNA template [27,44]. The completion of L1 integration requires elucidation, but likely involves host proteins involved in DNA repair and/or replication [45–48]. Notably, the L1-encoded proteins also can work *in trans* to retrotranspose other cellular RNAs such as Short Interspersed Elements (SINEs) (*e*.*g*., Alu [49] and SINE-R/VNTR/Alu (SVA) elements [50–52]). L1 also can mobilize uracil-rich small nuclear RNAs (*e*.*g*., U6 snRNA [48,53,54], small nucleolar RNAs (*e*.*g*., U3 snoRNA [55]), and messenger RNAs, which results in the formation of processed pseudogenes [35,36]).

Since L1 retrotransposition can be mutagenic, it stands to reason that the host cell employs multiple mechanisms to restrict L1 mobilization [reviewed in 56]. For example, cytosine methylation of the L1 5' UTR suppresses L1 expression [57,58]. In addition, piwi-interacting RNAs (piRNAs) suppress L1 expression in germ line cells [reviewed in 56, 59, and 60]. Finally, emerging studies have demonstrated that several cellular proteins restrict L1 retrotransposition. These proteins include several APOBEC3 family members [61, reviewed in 62], TREX1 [63], MOV10 [64–66], hnRNPL [67], SAMHD1 [68], RNase L [69], and the melatonin receptor 1 (MT1) [70].

To gain a more complete understanding of the interplay between the L1 retrotransposition machinery and the host cell, we used liquid chromatography-tandem mass spectrometry (LC-MS/MS) to identify proteins that co-immunoprecipitate with L1 ORF1p in HeLa cells, reasoning that some of these proteins may affect L1 retrotransposition. We next analyzed the effects of ORF1p-interacting proteins on L1 retrotransposition by overexpressing a subset of them in a cultured cell retrotransposition assay [30,71]. Here, we report that the zinc-finger antiviral protein ZAP [72] interacts with L1 RNPs and inhibits L1 retrotransposition in cultured cells. ZAP also inhibits human Alu retrotransposition and the retrotransposition of mouse and zebrafish LINE elements. Molecular genetic and biochemical analyses suggest that ZAP inhibits retrotransposition by suppressing the accumulation of full-length L1 RNA and L1-encoded proteins in the cell.

## Results

### Identification of L1 ORF1p-interacting proteins

**Fig 1:**
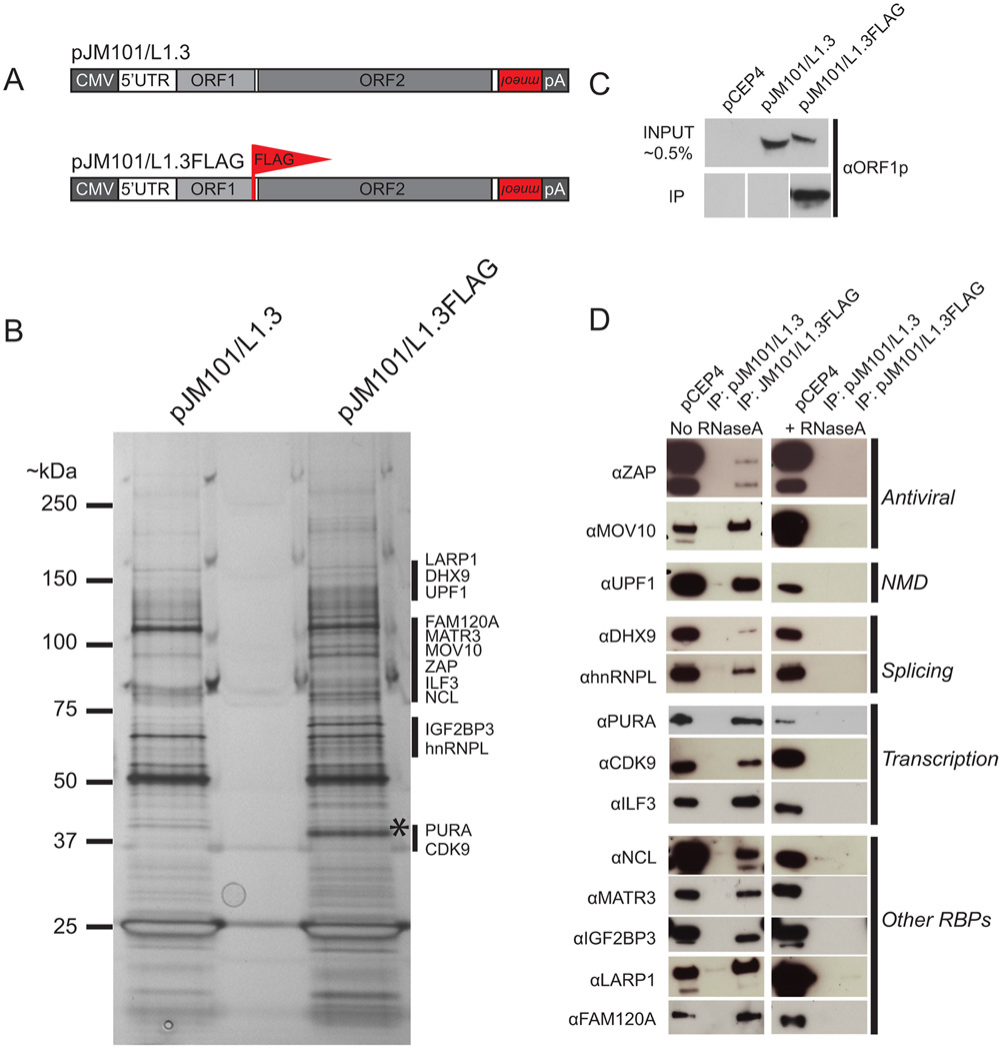
The identification of host proteins immunoprecipitated with L1 ORF1p-FLAG. *(A) Schematic of L1 constructs*: pJM101/L1.3 expresses a human L1 (L1.3) [5] containing an *mneoI* retrotransposition indicator cassette within the L1 3' UTR. The pJM101/L1.3FLAG construct is identical to pJM101/L1.3, but contains a single FLAG epitope on the carboxyl-terminus of ORF1p. Both constructs were cloned into a pCEP4 mammalian expression vector. A CMV promoter augments L1 expression and an SV40 polyadenylation signal (pA) is located downstream of the native L1 polyadenylation signal. *(B) Results of immunoprecipitation experiments*: Whole cell lysates from HeLa cells transfected with either pJM101/L1.3 or pJM101/L1.3FLAG were subjected to immunoprecipitation using an anti-FLAG antibody. The proteins then were separated by SDS-PAGE, visualized by silver staining, and subjected to LC-MS/MS. An ~40 kDa band corresponding to the theoretical molecular weight of ORF1p is visible in the pJM101/L1.3FLAG lane (*). Black bars indicate the approximate molecular weights of the ORF1p-FLAG interacting proteins. Molecular weight standards (kDa) are shown on the left hand side of the gel. *(C) Validation of the ORF1p-FLAG immunoprecipitation*: Western blot experiments using an antibody specific to amino acids 31–49 of L1.3 ORF1p verified the enrichment of ORF1p-FLAG in pJM101/L1.3FLAG, but not pJM101/L1.3 immunoprecipitation reactions. Cells transfected with the pCEP4 vector served as a negative control. *(D) Validation of putative ORF1p-FLAG interacting proteins*: Western blot images of the pJM101/L1.3FLAG and pJM101/L1.3 immunoprecipitation (IP) reactions. The pCEP4 lanes denote whole cell lysates derived from HeLa cells transfected with an empty pCEP4 vector (~ 1.0% input). Primary antibodies used to probe western blots are indicated to the left of the images. Immunoprecipitation reactions were conducted in either the absence (left) or presence (right) of RNaseA (10 μg/mL). The putative cellular functions of the ORF1p-FLAG interacting proteins are indicated on the right hand side of the blots.

To identify proteins that interact with L1 ORF1p, we transfected HeLa cells with a human L1 construct, pJM101/L1.3FLAG, which expresses a version of ORF1p containing a FLAG epitope at its carboxyl-terminus (ORF1p-FLAG) (Fig 1A). The pJM101/L1.3FLAG construct exhibits robust retrotransposition activity in HeLa cells, albeit at a lower efficiency (~50%) than the untagged L1 construct, pJM101/L1.3 (S1A Fig).

Briefly, HeLa cells were transfected with pJM101/L1.3FLAG or pJM101/L1.3, a similar construct that lacks the FLAG epitope sequence (Fig 1A). Whole cell lysates from transfected cells then were incubated with anti-FLAG coated agarose beads to immunoprecipitate ORF1p-FLAG (see Methods). Immunoprecipitated fractions were analyzed by SDS-PAGE and proteins were visualized by silver staining (Fig 1B). The analysis of silver-stained gels revealed a prominent band of ~40 kDa (the theoretical molecular weight of ORF1p) in the pJM101/L1.3FLAG immunoprecipitation lane (Fig 1B; asterisk), which was not apparent in the pJM101/L1.3 lane. Western blot analysis with an antibody specific to L1.3 ORF1p (amino acids 31–49) confirmed the enrichment of ORF1p-FLAG in the pJM101/L1.3FLAG lane (Fig 1C and S1B Fig; bottom panel). We also observed a complex pattern of bands between ~25 kDa and ~150 kDa that was present in the pJM101/L1.3FLAG lane that was not evident in the pJM101/L1.3 lane (Fig 1B; black vertical bars). A similar pattern of protein bands was produced on silver-stained gels from pJM101/L1.3FLAG immunoprecipitation reactions using different wash and/or lysis conditions (S1B and S1C Fig, respectively).

To determine the identity of cellular proteins that associated with ORF1p-FLAG, the bands from the lanes corresponding to the pJM101/L1.3FLAG and pJM101/L1.3 immunoprecipitation experiments were excised from SDS-PAGE gels and submitted for LC-MS/MS (see Methods). An LC-MS/MS-identified protein was selected as an ORF1p-interacting candidate if it met the following criteria: 1) the protein was unique to the pJM101/L1.3FLAG immunoprecipitation, and 2) the protein was identified by two or more unique peptide sequences (peptide error rate ≤0.05; protein probability ≥0.95) (S1 Table and Methods). Thirty-nine ORF1p-interacting protein candidates were identified that met these criteria (S1 Table).

To confirm the interactions between LC-MS/MS-identified proteins and ORF1p-FLAG, we evaluated 13 of the 39 ORF1p-FLAG interacting proteins for which there were commercially available antibodies and/or cDNA expression clones. Western blot analyses confirmed that these proteins associated with ORF1p-FLAG (Fig 1D). The 13 ORF1p-interacting proteins are involved in a variety of cellular processes including antiviral defense (ZAP [72] and MOV10 [73]), nonsense-mediated decay (UPF1 [74]), RNA splicing (hnRNPL [75] and DHX9 [76,77]), and transcription (PURA [78], CDK9 [79], and ILF3 [80]). Notably, gene ontology [81] and global analyses of RNA binding proteins in human cell lines [82,83] revealed that the 13 validated ORF1p-FLAG interacting proteins are RNA binding proteins (RBPs). Consistently, immunoprecipitation experiments of ORF1p-FLAG conducted in the presence of RNaseA disrupted the association between ORF1p and each of the 13 ORF1p-interacting proteins (Fig 1D). Thus, the majority of ORF1p-interacting proteins associate with ORF1p by binding to L1 RNA and/or other RNAs present within the L1 RNP [84].

### ORF1p-interacting proteins restrict L1 retrotransposition in HeLa cells

**Fig 2:**
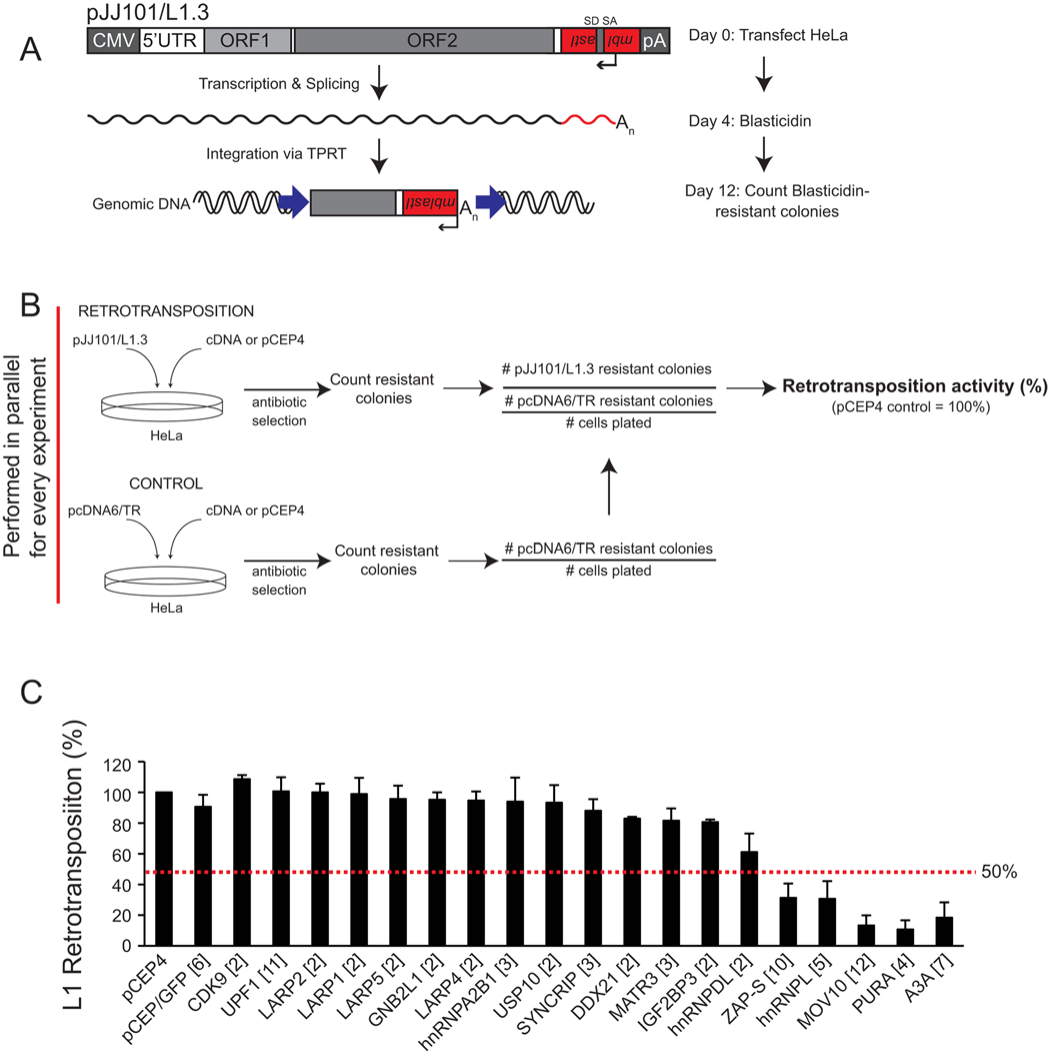
Several of the ORF1p-FLAG interacting proteins inhibit L1 retrotransposition. *(A) Schematic of the cultured-cell retrotransposition assay*: HeLa cells were transfected with an engineered human L1.3 construct (pJJ101/L1.3) marked with a blasticidin indicator cassette (*mblastI*). The pJJ101/L1.3 construct was cloned into a pCEP4 mammalian expression vector. A CMV promoter augments L1 expression and an SV40 polyadenylation signal (pA) is located downstream of the native L1 polyadenylation signal. The *mblastI* cassette is cloned into the L1 3' UTR antisense to the L1 and contains a blasticidin deaminase gene that is disrupted by an intron in the L1 sense orientation. The blasticidin deaminase gene can only be expressed when the L1 transcript is spliced, reverse transcribed, and inserted into genomic DNA [30,71]. *(B) Schematic of the pJJ101/L1*.*3 retrotransposition screen*: To analyze the effect of the ORF1p-FLAG interacting proteins on L1 retrotransposition, HeLa cells were co-transfected with equal amounts of pJJ101/L1.3 and a cDNA plasmid expressing one of the candidate ORF1p-FLAG interacting proteins or a pCEP4 empty vector. To control for potential off-target effects, HeLa cells also were co-transfected with a control plasmid (pcDNA6/TR) that expresses the blasticidin deaminase gene and a cDNA plasmid expressing one of the candidate proteins or a pCEP4 empty vector. Both assays were subjected to the same blasticidin selection regimen. The resultant number of blasticidin-resistant colonies in pcDNA6/TR control assays provides a visual, quantitative readout of the effect of cDNA overexpression on the ability of cells to grow in the presence of blasticidin. *(C) Results of pJJ101/L1*.*3 retrotransposition screen*: HeLa cells were co-transfected with pJJ101/L1.3 and each of the indicated cDNA expressing plasmids. L1 retrotransposition was assayed in 6-well tissue culture plates. The X-axis indicates the cDNA that was co-transfected with pJJ101/L1.3. The bracketed number next to each cDNA indicates the number of independent experiments. The Y-axis indicates L1 retrotransposition activity after accounting for cDNA toxicity (see Fig 2B). Retrotransposition activity (black bars) is normalized to the pCEP4 empty vector control. Error bars represent the standard deviation for each set of experiments. The red dotted line indicates a 50% inhibition of retrotransposition activity.

We next investigated whether overexpression of nine of the validated ORF1p-interacting proteins, as well as nine unvalidated ORF1p-interacting proteins, affects L1 retrotransposition [30,71]. Briefly, HeLa cells were co-transfected with a cDNA plasmid expressing one of the ORF1p-FLAG interacting proteins and an engineered human L1 construct (pJJ101/L1.3; [85]) marked with a blasticidin retrotransposition indicator cassette (*mblastI*) (Fig 2A and 2B; top panel). The *mblastI* cassette contains an antisense copy of the blasticidin deaminase gene, which is cloned into the L1 3' UTR. The blasticidin deaminase gene also is interrupted by an intron in the same transcriptional orientation as L1. This arrangement ensures that the blasticidin deaminase gene is expressed only when the L1 transcript is spliced, reverse transcribed, and inserted into genomic DNA. The resulting blasticidin-resistant foci then provide a visual, quantitative readout of retrotransposition activity [30,45].

To monitor potentially toxic side effects of cDNA overexpression, HeLa cells also were co-transfected in a parallel assay with a cDNA expression vector and a control plasmid (pcDNA6/TR) that expresses the blasticidin deaminase gene (Fig 2B; bottom panel). Following blasticidin selection, the resulting foci provide a visual, quantitative readout of the effect of cDNA overexpression on colony formation (Fig 2B; bottom panel). This control is essential to determine if a cDNA affects L1 retrotransposition or cell viability and/or growth.

We co-transfected HeLa cells with each of the 18 ORF1p-FLAG interacting candidates and pJJ101/L1.3 (Fig 2C). An empty pCEP4 vector that was co-transfected with pJJ101/L1.3 served as a normalization control (Fig 2B and 2C). As a negative control, we demonstrated that a plasmid that expresses the humanized renilla green fluorescence protein (pCEP/GFP) did not affect pJJ101/L1.3 retrotransposition. As a positive control, we demonstrated that a plasmid that expresses human APOBEC3A (pK_A3A) reduced pJJ101/L1.3 retrotransposition to ~18% of control levels (Fig 2C), which is in agreement with previous studies [61,86–88]. Four of the cDNA-expressing plasmids that we tested (ZAP-S (ZAP short isoform), hnRNPL, MOV10, and PURA) each reduced pJJ101/L1.3 retrotransposition to less than 50% of pCEP4 control levels. Notably, ZAP-S (~30% of control), hnRNPL (~30% of control), MOV10 (~13% of control), and PURA (~10% of control) inhibited retrotransposition to levels similar to that of pK_A3A (~18% of control) (Fig 2C). By comparison, the majority of the cDNA-expressing plasmids (14/18) did not significantly affect pJJ101/L1.3 retrotransposition levels (less than 50% inhibition when compared to pCEP4 control levels) (Fig 2C). Thus, the data suggest that ZAP-S, hnRNPL, MOV10, and PURA inhibit L1 retrotransposition in cultured cells.

### ZAP inhibits L1 retrotransposition

The above data (Fig 2C) imply that ZAP, hnRNPL, MOV10, and PURA may function as host factors that restrict L1 retrotransposition. Notably, hnRNPL [67], MOV10 [64,65], and PURA [89] previously were shown to inhibit L1 retrotransposition. However, the effect of ZAP on L1 retrotransposition has not been studied; thus, we sought to determine how ZAP inhibits L1 retrotransposition.

**Fig 3:**
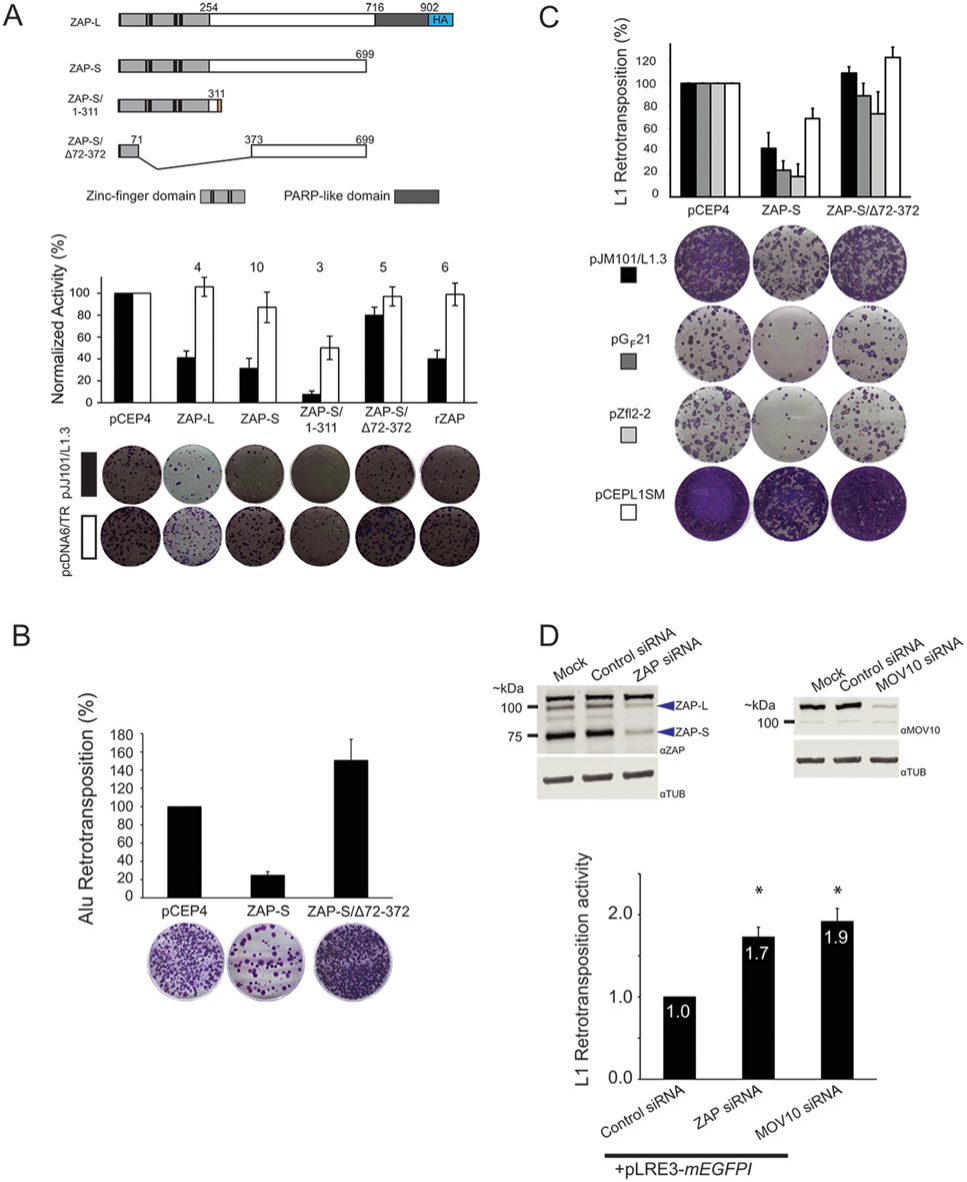
ZAP-S inhibits LINE and Alu retrotransposition. *(A) ZAP inhibits L1 retrotransposition*: Top panel: Schematics of ZAP constructs. Depicted are the relative positions of the zinc-finger domains (light gray rectangles), cysteine-histidine (CCCH) zinc-fingers (vertical black bars), and PARP-like domain (dark gray rectangles) of the ZAP-L and ZAP-S expression constructs. ZAP-L contains a carboxyl-terminal HA tag (blue rectangle labeled HA). The ZAP-S/1-311 construct contains an additional 31 amino acids at the carboxyl terminus. The ZAP-S/∆72–372 harbors a deletion that removes the CCCH zinc fingers (See Methods). Middle panel: Results of the retrotransposition assays. The X-axis indicates the cDNA co-transfected with pJJ101/L1.3 or pcDNA6/TR. The Y-axis indicates pJJ101/L1.3 retrotransposition activity (black bars), or pcDNA6/TR colony formation activity (white bars). All values have been normalized to the pCEP4 empty vector control (100%). The numbers above the bar graphs indicate the number of independent experiments performed with each cDNA expression construct. Error bars represent standard deviations. Bottom panel: A single well of a representative six-well tissue culture plate, displaying blasticidin-resistant colonies from the pJJ101/L1.3 retrotransposition assay (top, black rectangle) and the pcDNA6/TR control assay (bottom, white rectangle). *(B) ZAP inhibits Alu retrotransposition*: The X-axis indicates the cDNA co-transfected with pJM101/L1.3Δneo and pAlu*neo* ^Tet^. The Y-axis indicates the retrotransposition efficiency. All values are normalized to the pCEP4 empty vector control (100%). Control assays using a plasmid that expresses the neomycin phosphotransferase gene (pcDNA3) were conducted similarly to pcDNA6/TR control assays as outlined in Fig 2B. Representative images of G418-resistant HeLa foci from the Alu retrotransposition assay are shown below the bar graph. The results are the average of three independent experiments. Error bars indicate standard deviations. *(C) ZAP inhibits the retrotransposition of mouse and zebrafish LINE elements*. The X-axis indicates the cDNA that was co-transfected with human L1 (pJM101/L1.3 (black bars)), mouse L1 (pG_F_21 (dark grey bars)), zebrafish L2 (pZfL2-2 (light grey bars)), or synthetic mouse L1 (pCEPsmL1 (white bars)). The Y-axis indicates the retrotransposition efficiency. Representative images of G418-resistant HeLa cell foci are shown below the bar graph. Control assays using a plasmid that expresses the neomycin phosphotransferase gene (pcDNA3) were conducted similarly to pcDNA6/TR control assays outlined in Fig 2B. All values are normalized to the pCEP4 empty vector control (100%). Error bars indicate standard deviations. *(D) The depletion of ZAP enhances L1 retrotransposition*: Top panels: Western blots of whole cell lysates derived from mock HeLa cell transfections or HeLa cells transfected with indicated siRNAs. Blue arrows point to the approximate location of ZAP-L and ZAP-S. Bottom panel: The bar graph depicts pLRE-*mEGFP1* retrotransposition activity following siRNA treatment. The X-axis indicates the siRNA. The Y-axis indicates the pLRE-*mEGFP1* retrotransposition efficiency normalized to the control siRNA (set to 1). Retrotransposition efficiency values are reported as the mean from four independent experiments. Error bars indicate the standard deviations. Asterisks indicate statistically significant differences from the control siRNA experiments (two-tailed t test/p<0.05).

ZAP is a poly (ADP-ribose) polymerase (PARP) family member [90] initially characterized as an antiviral protein that inhibits murine leukemia virus (MLV) replication in cultured rat cells [72]. Previous studies identified two human ZAP isoforms that resulted from alternative splicing [90] (Fig 3A; top panel). The long ZAP isoform (ZAP-L) is 902 amino acids in length and contains an amino-terminus CCCH zinc-finger domain and an inactive carboxyl-terminal PARP-like domain [90]. The short ZAP isoform (ZAP-S) is 699 amino acids in length and lacks the carboxyl-terminal PARP-like domain [90]. The HA-tagged human ZAP-L isoform restricted pJJ101/L1.3 retrotransposition to ~40% of control levels (Fig 3A; black bars) and the human ZAP-S isoform restricted pJJ101/L1.3 retrotransposition to ~30% of control levels (Figs 2C and 3A; black bars). Notably, overexpression of ZAP-L or ZAP-S did not dramatically affect the ability of HeLa cells to form blasticidin-resistant colonies in pcDNA6/TR control assays (Fig 3A, white bars). Western blot control experiments confirmed the overexpression of ectopic ZAP-L and ZAP-S compared to untransfected controls ~48 hours post-transfection (S2A and S2B Fig). Thus, ZAP inhibits L1 retrotransposition in cultured cells and the ZAP-L PARP-like domain is not required for L1 restriction.

Putative ZAP orthologs are present in several species [90]; thus, we tested whether a rat ZAP cDNA (rZAP) [72], that is orthologous to human ZAP-S [72,90] could restrict pJJ101/L1.3 retrotransposition. Overexpression of rZAP efficiently reduced retrotransposition to ~40% of control levels (Fig 3A; black bars). Thus, the ability to restrict L1 retrotransposition is not limited to human ZAP.

### The ZAP zinc-finger domain is necessary and sufficient to inhibit L1 retrotransposition

The ZAP zinc-finger domain binds to RNA and is required for antiviral activity [91,92]. To analyze the role of the ZAP zinc-finger domain in L1 restriction, we tested the effects of a truncated ZAP-S mutant that expresses the ZAP zinc-finger domain (ZAP-S/1-311; containing amino acids 1–311) as well as a ZAP-S mutant that lacks the zinc-finger domain (ZAP-S/Δ72–372; lacking amino acids 72–372) in pJJ101/L1.3 retrotransposition assays (Fig 3A; above graph). ZAP-S/1-311 restricted retrotransposition to ~10% of control levels (Fig 3A; black bars), whereas ZAP-S/Δ72–372 had little effect on retrotransposition (~80% of control levels) (Fig 3A; black bars). The overexpression of the wild type or mutant ZAP-S/Δ72–372 expression constructs did not adversely affect the ability of HeLa cells to form blasticidin-resistant colonies in pcDNA6/TR control assays (Fig 3A; white bars). Notably, transfection with ZAP-S/1-311 resulted in an ~50% decrease in the ability of HeLa cells to form blasticidin-resistant colonies; however, this effect has been accounted for through normalization (Fig 2B) and thus is independent of the ability of ZAP-S/1-311 to restrict L1 retrotransposition. Indeed, similar off-target effects have been reported for A3A cDNA expressing plasmids in HeLa cell-based L1 retrotransposition assays [61]. Western blot control experiments revealed that wild-type ZAP-S and the two mutant ZAP-S isoforms were expressed at similar levels ~48 hours post-transfection (S2B and S2C Fig). Thus, the ZAP zinc-finger domain is necessary and sufficient to inhibit L1 retrotransposition.

### ZAP restricts the retrotransposition of various non-LTR retrotransposons

To determine if ZAP-S was able to restrict other non-long terminal repeat (non-LTR) retrotransposons, we tested whether ZAP-S expression affected human Alu retrotransposition. Unlike L1, Alu is a 7SL-derived non-autonomous retrotransposon that does not encode its own proteins [93]. Instead, Alu elements must parasitize L1 ORF2p *in trans* to mediate their retrotransposition [49]. Briefly, HeLa cells were co-transfected with a full-length L1 element (pJM101/L1.3Δneo), an Alu retrotransposition reporter plasmid (pAlu*neo* ^Tet^), and a ZAP-S expression plasmid. Notably, ZAP-S potently reduced Alu retrotransposition to ~25% of control levels (Fig 3B). In contrast, the expression of the L1 restriction-deficient ZAP-S/Δ72–372 mutant did not negatively affect Alu retrotransposition (Fig 3B). Thus, ZAP-S is able to restrict the mobility of the two most prolific retrotransposons present in the human genome.

We next tested if human ZAP-S could restrict the retrotransposition of a natural mouse L1 (pG_F_21) [94], a zebrafish LINE-2 (pZfL2-2) [95], or a synthetic mouse L1 (pCEPsmL1) [96] that has been extensively mutagenized to alter 24% of the nucleic acid sequence without disrupting amino acid sequence. Human ZAP-S inhibited the retrotransposition of human L1 (pJM101/L1.3; ~43% of control levels), natural mouse L1 (pG_F_21; ~24% of control levels), zebrafish L2 (pZfL2-2; ~19% of control levels), and synthetic mouse L1 (pCEPsmL1; ~70% of control levels) (Fig 3C). The restriction-defective ZAP-S mutant, ZAP-S/Δ72–372, did not significantly affect the retrotransposition activity of these retrotransposons (Fig 3C). Notably, the milder inhibition of ZAP-S on pCEPsmL1 may be due to the elevated efficiency of pCEPsmL1 retrotransposition, the increased steady-state level of pCEPsmL1 mRNA and proteins, and/or the GC-rich nature of pCEPsmL1 [96]. Thus, ZAP-mediated restriction of retrotransposition is not specific to human non-LTR retrotransposons.

### Depletion of endogenous ZAP enhances L1 retrotransposition

To test if endogenous ZAP restricts L1 retrotransposition, we used small interfering RNA (siRNA) to deplete endogenous ZAP from HeLa cells. Following siRNA treatment, cells were transfected with an L1 plasmid (pLRE3-*mEGFPI*) tagged with an *EGFP* indicator cassette (*mEGFPI*), which allows retrotransposition activity to be detected by EGFP fluorescence [97]. As a negative control, HeLa cells were transfected with the L1 retrotransposition-defective plasmid pJM111-LRE3-*mEGFPI*, which carries two missense mutations that adversely affect ORF1p RNA binding [22,30,98]. Treatment of HeLa cells with an siRNA pool against ZAP resulted in an ~80% and ~ 90% reduction of ZAP-L and ZAP-S protein levels, respectively, when compared to HeLa cells treated with a non-targeting control siRNA pool (Fig 3D; top left panel). ZAP siRNA treatment led to an approximately two-fold increase in pLRE3-*mEGFPI* retrotransposition activity when compared to assays conducted in the presence of a control siRNA (Fig 3D; bottom panel and S2D Fig). We further demonstrated that siRNA-mediated depletion of endogenous MOV10 (Fig 3D; top right panel) from HeLa cells resulted in an approximately two-fold increase in pLRE3-*mEGFPI* retrotransposition (Fig 3D; bottom panel and S2D Fig), which is in agreement with previous studies [64,65]. These data suggest that endogenous ZAP may restrict L1 retrotransposition.

### ZAP-S inhibits the accumulation of full-length LINE-1 mRNA

**Fig 4:**
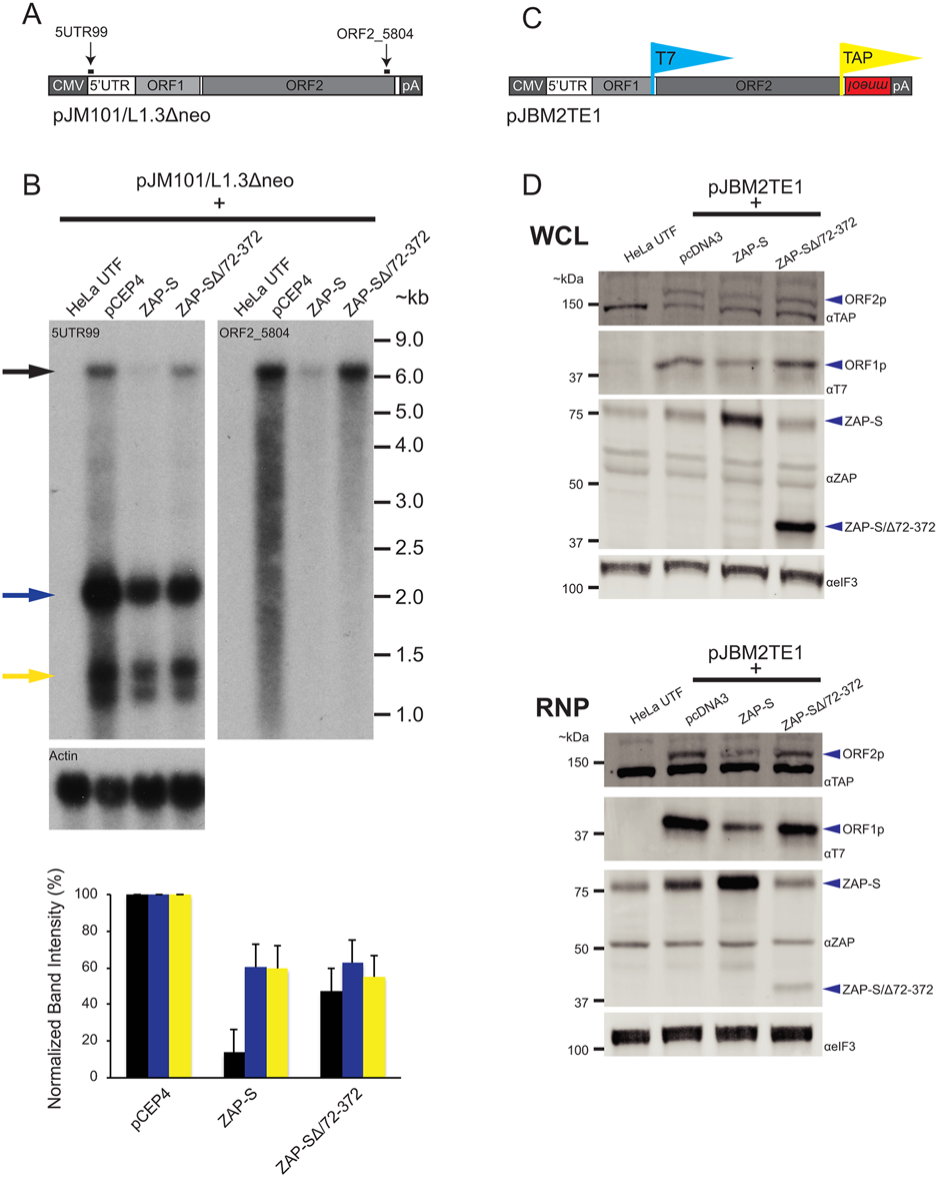
The effect of ZAP-S on L1 RNA and L1 protein expression. *(A) Schematic of pJM101/L1*.*3Δneo*: Bold black lines indicate the approximate location of probes (5UTR99 and ORF2_5804) used in the northern blot experiments. pJM101/L1.3Δneo is expressed from a pCEP4 vector. A CMV promoter augments L1 expression and an SV40 polyadenylation signal (pA) is located downstream of the native L1 polyadenylation signal. *(B) Results of northern blots*: Top panel: HeLa cells were co-transfected with pJM101/L1.3Δneo and either the indicated ZAP-S expression plasmids or an empty pCEP4 vector. Northern blot images depict the effect of ZAP-S overexpression on polyadenylated L1 RNA levels. The constructs transfected into HeLa cells are indicated above each lane. UTF indicates untransfected HeLa cells and serves as a negative control. Probes (5UTR99 and ORF2_5804) are indicated in the top left corner of the respective blots. The black arrow indicates the position of the full-length L1 RNA. The blue and yellow arrows indicate shorter L1 RNA species. The experiment was repeated three times with similar results. Actin served as a loading control. RNA size standards (~kb) are shown at the right of the blot image. Bottom panel: Quantification of northern blot bands. The X-axis indicates the cDNA expression construct that was co-transfected with pJM101/L1.3Δneo. The Y-axis indicates relative band intensity normalized to pCEP4 controls (100%). Black bars represent the full-length L1 band. Blue and yellow bars represent the smaller L1 RNA bands, corresponding to the colored arrows, respectively, in the top panel. The results are the average of three independent experiments. Error bars represent standard deviations. *(C) Schematic of pJBM2TE1*: The construct contains a T7 epitope tag on the carboxyl-terminus of ORF1p and a TAP tag on the carboxyl-terminus of ORF2p. An *mneoI* retrotransposition indicator cassette is present in the 3’ UTR. pJMB2TE1 is expressed from a pCEP4 backbone, which has been modified to contain a puromycin selectable marker. A CMV promoter augments L1 expression and an SV40 polyadenylation signal (pA) is located downstream of the native L1 polyadenylation signal. *(D) ZAP-S decreases the accumulation of the L1-encoded proteins*: HeLa cells were co-transfected with pJBM2TE1 and the plasmids indicated above each lane. UTF indicates untransfected HeLa cells and serves as a negative control. Depicted are western blots using whole cell lysates (WCL, top panel) or RNP fractions (RNP, bottom panel). Blue arrows indicate the positions of ORF2p, ORF1p, ZAP-S, and ZAP-S/∆72–372. The eIF3 protein is used as a loading control. Representative images are shown. The experiments were repeated three times with similar results.

To investigate how ZAP restricts L1 retrotransposition, we analyzed the effect of ZAP-S expression on the accumulation of the L1 RNA. HeLa cells were co-transfected with pJM101/L1.3Δneo and either ZAP-S or ZAP-S/Δ72–372. Polyadenylated RNA from whole cell extracts then was analyzed by northern blot using RNA probes complementary to sequences within the L1.3 5' UTR (5UTR99) and ORF2 (ORF2_5804) (Fig 4A). Co-transfection with ZAP-S resulted in a reduction of full-length polyadenylated L1 RNA levels (~13% of pCEP4 control) compared to cells co-transfected with either the restriction-defective ZAP-S/Δ72–372 (~47% compared to pCEP4 control) or an empty pCEP4 control vector (Fig 4B; black arrow in blot; black bars in graph). Interestingly, ZAP-S expression did not have a pronounced effect on the accumulation of smaller L1 RNA species, which may have resulted from cryptic splicing and/or premature polyadenylation (Fig 4B; top panel: blue and yellow arrows, bottom panel: blue and yellow bars) [99–101]. Finally, control experiments revealed that ectopic ZAP-S expression did not affect endogenous actin RNA levels (Fig 4B). Thus, ZAP-S expression reduces the accumulation of full-length L1 mRNA in cultured cells.

### ZAP-S inhibits the accumulation of ORF1p and ORF2p

We next examined the effect of ZAP-S expression on the accumulation of ORF1p and ORF2p. We co-transfected HeLa cells with either ZAP-S or ZAP-S/Δ72–372 and the L1 plasmid, pJBM2TE1, which expresses an L1.3 element marked with a T7 *gene10* epitope tag on the carboxyl-terminus of ORF1p and a TAP epitope-tag on the carboxyl-terminus of ORF2p (Fig 4C). Following co-transfection, HeLa cells were treated with puromycin to select for cells expressing pJBM2TE1. Both whole cell lysates (WCL) and RNP fractions were collected 5 days post-transfection and subjected to western blot analyses to monitor ORF1p and ORF2p expression levels.

Expression of ZAP-S led to a decrease in the level of ORF1p and ORF2p in both WCL and RNP fractions, whereas the expression of the restriction-defective ZAP-SΔ/72-372 mutant or an empty pcDNA3 vector did not dramatically affect ORF1p or ORF2p expression levels (Fig 4D). The reduction in ORF1p and ORF2p was most evident in the RNP fraction, likely because both ORF1p and ORF2p are enriched in RNPs [19,26,37,38,102]. Control experiments revealed that ZAP-S expression did not affect the level of eIF3 protein (Fig 4D) and that ZAP-S and ZAP-S/Δ72–372 are expressed at similar levels in whole cell lysates (Fig 4D: top WCL panel). By comparison, ZAP-SΔ/72-372 is present at much lower levels in the RNP fraction compared to wild-type ZAP-S (Fig 4D; bottom RNP panel), suggesting that the zinc-finger domain is responsible for ZAP-S localization to the RNP fraction.

To determine if ZAP-S affects the expression of non-L1 proteins, we examined the effect of ZAP-S on EGFP expression. We co-transfected ZAP-S with an L1 plasmid (pLRE3-EF1-*mEGFP*ΔIntron) [103] that expresses the L1 element, LRE3 and an intact copy of the EGFP gene (S3A Fig). In this case, LRE3 and EGFP are under the control of convergent promoters, which allows the simultaneous expression of LRE3 and EGFP from pLRE3-EF1-*mEGFP*ΔIntron. Thus, EGFP expression is not dependent on retrotransposition. Forty-eight hours post-transfection, flow cytometry was used to isolate EGFP-positive cells (*i*.*e*., cells expressing pLRE3-EF1-*mEGFP*ΔIntron) (S3C Fig). Western blotting demonstrated a marked reduction in ORF1p when compared to EGFP levels in cells that were co-transfected with ZAP-S (S3B Fig). By comparison, ORF1p and EGFP were present at comparable levels in cells that were co-transfected with either an empty pCEP4 vector or the restriction-deficient ZAP-SΔ/72-372 mutant (S3B Fig). Control experiments revealed that ZAP-S did not affect endogenous tubulin protein levels (S3B Fig). Thus, ZAP-S expression appears to preferentially restrict the expression of L1 ORF1p.

### ZAP co-localizes with ORF1p and L1 RNA in the cytoplasm

**Fig 5:**
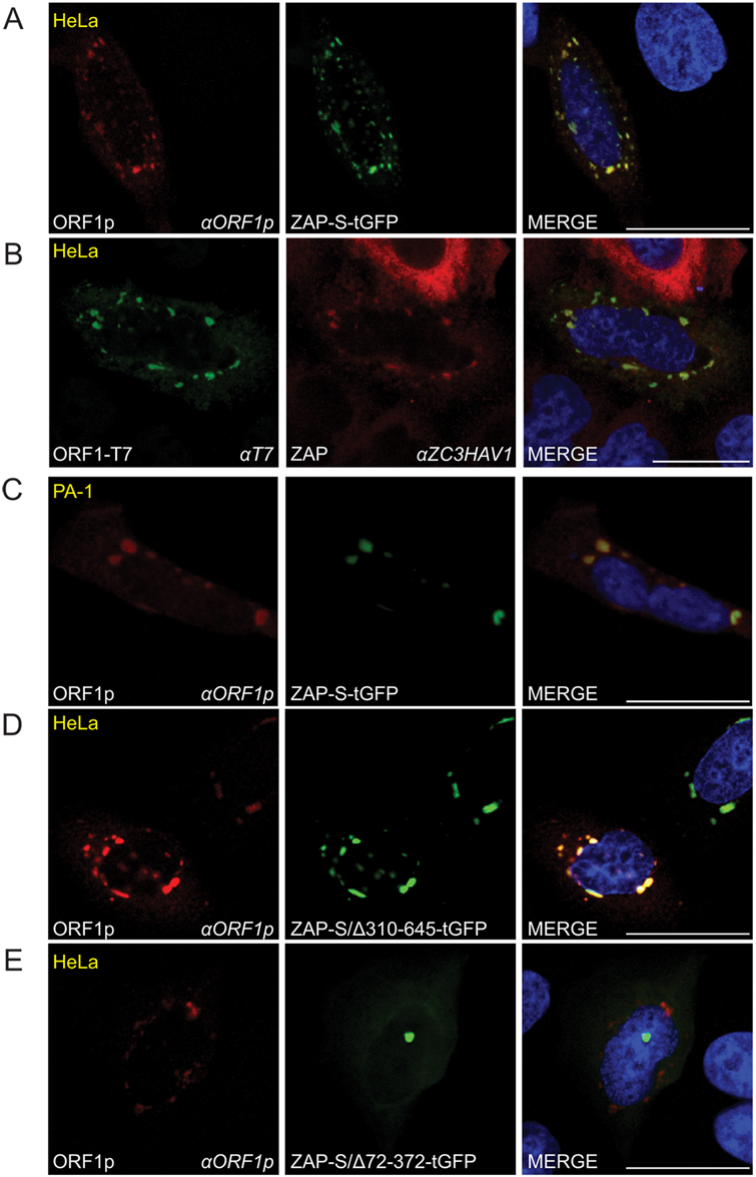
The co-localization of ORF1p and ZAP in cytoplasmic foci. *(A) Co-localization of transfected ORF1p and ZAP-S in HeLa cells*: ORF1p (red) expressed from pJM101/L1.3Δneo co-localizes with ZAP-S-tGFP (green). The experiment was repeated five times with similar results. *(B) Co-localization of transfected ORF1p with endogenous ZAP in cytoplasmic foci in HeLa cells*: ORF1p-T7 (green) expressed from pAD2TE1 co-localizes with endogenous ZAP (red). The experiment was repeated five times with similar results. *(C) Co-localization of transfected ZAP-S-tGFP with endogenous ORF1p in cytoplasmic foci in PA-1 cells*: ZAP-S-tGFP (green) co-localizes with endogenous ORF1p (red). PA-1 experiments were repeated twice with similar results. *(D-E) The ZAP-S zinc-finger domain is necessary for co-localization with ORF1p in HeLa cells*: ORF1p (red) expressed from pJM101/L1.3Δneo co-localizes with ZAP-S/Δ310-645-tGFP (green) (panel D). ORF1p (red) expressed from pJM101/L1.3Δneo forms cytoplasmic foci that do not contain ZAP-S/Δ72-372-tGFP (green) (panel E). The right-most image of each panel represents a merged image. The cell type is indicated at the top left (yellow), the protein name is listed on the bottom left, and the name of the primary antibody used (*italicized*) is annotated at the bottom right. Nuclei were stained with DAPI (blue) and the scale bar represents 25 μM.

ORF1p, ORF2p, and L1 RNA form RNP complexes that appear as discrete cytoplasmic foci when visualized by fluorescence microscopy [25,26,104]. Notably, previous studies have shown that ZAP predominantly is localized in the cytoplasm [105] and that ZAP antiviral activity also is localized to the cytoplasm [72]. To determine if ZAP co-localizes with ORF1p, we co-transfected HeLa cells with pJM101/L1.3Δneo and a plasmid that expresses a carboxyl-terminus turbo-GFP tagged ZAP-S protein (ZAP-S-tGFP). Control experiments showed that ZAP-S-tGFP restricted pJJ101/L1.3 retrotransposition to ~55% of control levels (S4A Fig). Confocal fluorescence microscopy revealed that ORF1p and ZAP-S-tGFP co-localized in discrete cytoplasmic foci in ~68% of cells that co-expressed both ORF1p and ZAP-S-tGFP (Fig 5A). To test if transfected ORF1p co-localizes with endogenous ZAP, we transfected HeLa cells with pAD2TE1, which expresses a human L1 (L1.3) containing a T7 *gene10* epitope-tag on the carboxyl-terminus of ORF1p [26]. Confocal microscopy revealed that endogenous ZAP co-localized with ORF1p-T7 in cytoplasmic foci in ~91% of cells that contained ORF1p-T7 foci (Fig 5B). Next, to test if endogenous ORF1p co-localizes with transfected ZAP-S, we transfected PA-1 cells (a human embryonic carcinoma-derived cell line that expresses endogenous ORF1p [106,107]) with ZAP-S-tGFP. Confocal microscopy demonstrated that endogenous ORF1p co-localized with ZAP-S-tGFP in ~89% of PA-1 cells that expressed ZAP-S-tGFP foci (Fig 5C). Thus, ORF1p and ZAP generally localize to the same region of the cytoplasm.

To test if the ZAP-S zinc-finger domain is critical for the co-localization of ZAP-S with ORF1p, we co-transfected HeLa cells with pJM101/L1.3Δneo and a tGFP-tagged ZAP-S mutant that expresses the ZAP-S zinc-finger domain (ZAP-S/Δ310-645-tGFP; lacking amino acids 310–645), or a ZAP-S mutant that lacks the ZAP-S zinc-finger domain (ZAP-S/Δ72-372-tGFP; lacking amino acids 72–372) (S4A Fig). In control experiments, ZAP-S/Δ310-645-tGFP restricted pJJ101/L1.3 retrotransposition to ~32% of control levels whereas ZAP-S/Δ72-372-tGFP did not have a significant effect (~93% of control) on retrotransposition activity (S4A Fig). Confocal microscopy revealed that ORF1p and ZAP-S/Δ310-645-tGFP co-localized in cytoplasmic foci in ~74% of cells that co-expressed both ORF1p and ZAP-S/Δ310-645-tGFP (Fig 5D). In cells transfected with pJM101/L1.3Δneo and ZAP-S/Δ72-372-tGFP, ORF1p and ZAP-S/Δ72-372-tGFP co-localized in only ~14% of cells that co-expressed both ORF1p and ZAP-S/Δ72-372-tGFP (Fig 5C). Thus, the ZAP-S zinc-finger domain is necessary and sufficient for the co-localization of ZAP-S and ORF1p in cytoplasmic foci.

**Fig 6:**
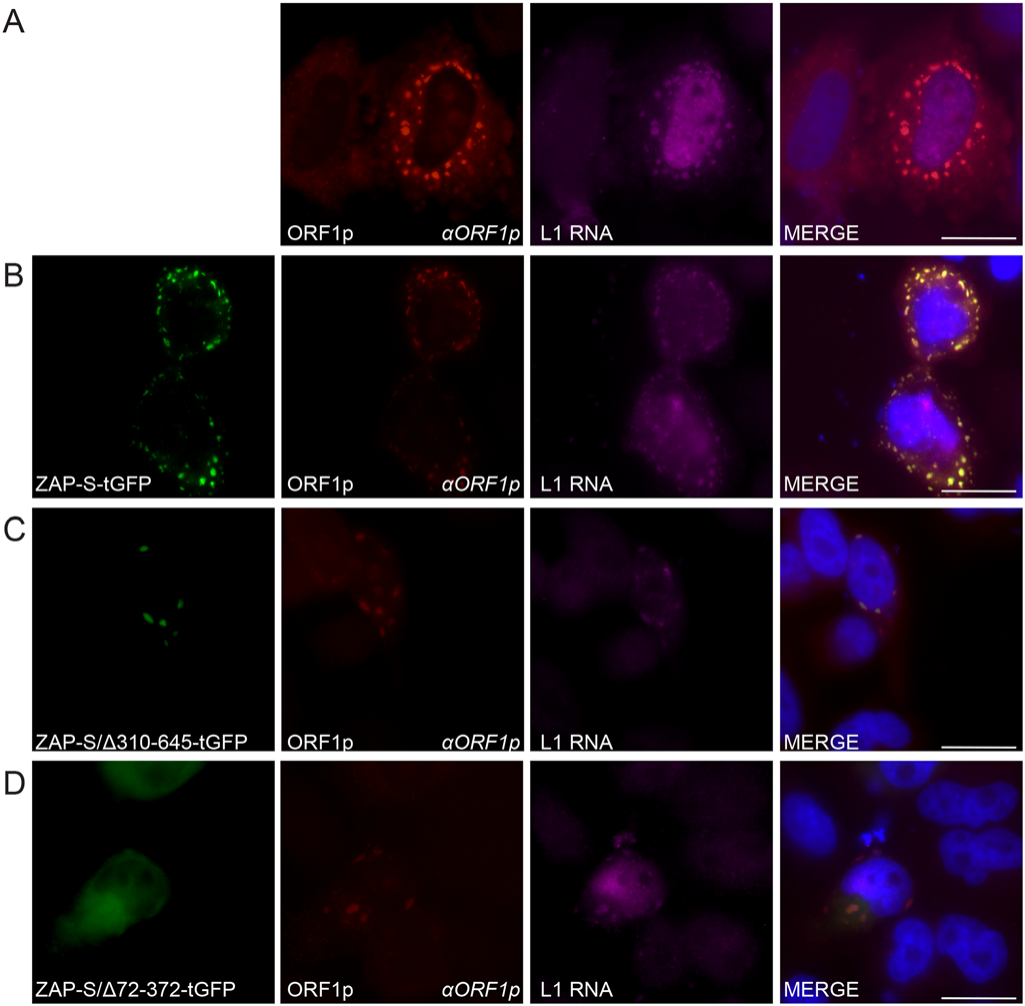
The co-localization of ZAP with L1 RNA and ORF1p in HeLa cells. *(A) Co-localization of transfected L1 RNA and ORF1p*: ORF1p (red) expressed from pJM101/L1.3 co-localizes with L1 RNA (magenta). *(B) Co-localization of transfected L1 RNA and ORF1p with transfected ZAP-S-tGFP in cytoplasmic foci*: ORF1p (red) and L1 RNA (magenta) expressed from pJM101/L1.3 co-localize with ZAP-S-tGFP (green). *(C-D) The ZAP-S zinc-finger domain is necessary for co-localization with ORF1p*: ORF1p (red) and L1 RNA (magenta) expressed from pJM101/L1.3 co-localize with ZAP-S/Δ310-645-tGFP (green) (panel C). ZAP-S/Δ72-372-tGFP (green) diffusely distributes throughout the cytoplasm, while ORF1p (red) expressed from pJM101/L1.3 forms cytoplasmic foci with L1 RNA (magenta) (panel D). The right-most image of each panel represents a merged image. The name of the protein or RNA is indicated at the bottom left, and the name of the primary antibody used (*italicized*) is annotated at the bottom right of each image. Nuclei were stained with DAPI (blue) and the scale bar represents 25 μM. Experiments were repeated three times with similar results.

To determine if ZAP co-localizes with L1 RNA, we co-transfected HeLa cells with pJM101/L1.3 and either ZAP-S-tGFP, ZAP-S/Δ310-645-tGFP, or ZAP-S/Δ72-372-tGFP. To visualize L1 RNA, transfected cells were probed with fluorescently labeled oligonucleotide probes complementary to sequences within the L1 5' UTR. As a control, cells were co-transfected with pJM101/L1.3 and an empty pCEP4 vector. In pCEP4 control experiments, fluorescence microscopy revealed that ORF1p co-localized with L1 RNA in cytoplasmic foci in ~88% of cells that contained ORF1p cytoplasmic Foci (Fig 6A and S5A Fig). In HeLa cells co-transfected with pJM101/L1.3 and ZAP-S-tGFP, L1 RNA co-localized with ORF1p and ZAP-S-tGFP in cytoplasmic foci in ~23% of foci-containing cells (Fig 6B and S5A Fig). Thus, ZAP and L1 RNA co-localize in cytoplasmic foci.

Fluorescence microscopy further revealed that in cells co-transfected with pJM101/L1.3 and ZAP-S/Δ310-645-tGFP that L1 RNA was detected in ORF1p and ZAP-S/Δ310-645-tGFP foci in only ~18% of foci-containing cells (Fig 6C and S5A Fig). In contrast, in cells co-transfected with pJM101/L1.3 and ZAP-S/Δ72-372-tGFP, L1 RNA co-localized with ORF1p in ~77% of cells that expressed ZAP/Δ72-372-tGFP and contained ORF1p cytoplasmic Foci (Fig 6D and S5A Fig). Thus, the data suggest that ZAP prevents the accumulation of L1 RNA in cytoplasmic foci.

We next determined the effect of ZAP-S on ORF1p expression using confocal microscopy. HeLa cells were co-transfected with pJM101/L1.3Δneo and either ZAP-S-tGFP, ZAP-S/Δ310-645-tGFP, or ZAP-S/Δ72-372-tGFP. As a control, cells were co-transfected with pJM101/L1.3Δneo and an empty pCEP4 vector. In pCEP4 control experiments, confocal microscopy revealed that ~10.8% of cells expressed ORF1p after ~ 48 hours (S5B Fig). In contrast, only ~2.8% of cells that were co-transfected with ZAP-S-tGFP expressed ORF1p and ~2.8% of cells that were co-transfected with ZAP-S/Δ310-645-tGFP expressed ORF1p (S5B Fig). Approximately 8.0% of cells that were co-transfected with ZAP-S/Δ72-372-tGFP expressed ORF1p (S5B Fig). Thus, the data suggest that the ZAP-S zinc-finger domain is necessary and sufficient to inhibit the accumulation of ORF1p in HeLa cells.

L1 cytoplasmic foci also co-localize with an array of RNA binding proteins, including markers of cytoplasmic stress granules (SGs) [26,89,104]. Notably, ZAP also localizes to cytoplasmic SGs [108]. To determine whether ZAP-S/ORF1p foci co-localize with cytoplasmic SGs we transfected HeLa cells with pJM101/L1.3Δneo and ZAP-S-tGFP. Confocal microscopy revealed that ZAP-S-tGFP/ORF1p co-localized with the endogenous SG associated protein eIF3 (S4B Fig). Additionally, endogenous ZAP also co-localized with the SG marker, G3BP (S4D Fig). In contrast, ZAP-S-tGFP/ORF1p foci did not co-localize with endogenous tubulin (S4C Fig), and endogenous ZAP did not co-localize with the processing body associated protein, DCP1α (S4E Fig). Thus, L1 ORF1p, ZAP, and SG associated proteins partition to the same cytoplasmic compartment.

## Discussion

In this study, we identified 39 cellular proteins that interact with L1 ORF1p and validated 13 of these interactions in biochemical assays. Our data showed that the 13 validated ORF1p-interacting proteins associate with ORF1p via an RNA bridge (Fig 1D). Notably, 33 out of 39 of the ORF1p-interacting proteins also were detected in recent studies (S1 Table; [47,67,89]). Importantly, we discovered that ZAP restricts human L1 and Alu retrotransposition. We also showed that hnRNPL, MOV10, and PURA inhibit L1 retrotransposition, which is in agreement with previous studies [64–67,89]. Thus, our data both confirm and extend those previous analyses and will help guide future studies that endeavor to determine how L1 retrotransposition impacts the human genome.

ZAP inhibits the mobility of both human and non-human non-LTR retrotransposons. The overexpression of the human and rat orthologs of ZAP restricts human L1 retrotransposition (Fig 3A). Human ZAP-S overexpression restricts the retrotransposition of an engineered human SINE (Alu) (Fig 3B), an engineered mouse L1 (G_F_21), and an engineered zebrafish LINE-2 element (ZfL2-2) (Fig 3C). Although our studies primarily involved the overexpression of ZAP, we also demonstrated that the depletion of endogenous ZAP in HeLa cells led to an ~2-fold increase in L1 retrotransposition (Fig 3D). This observed increase in L1 retrotransposition activity is similar to increases in L1 activity that were observed upon depletion of MOV10 and hnRNPL proteins in other studies [64,65,67]. Thus, in principle, physiological levels of ZAP may be sufficient to influence retrotransposition in certain cell types.

The ZAP CCCH zinc-finger domain is required to both bind and mediate the degradation of viral RNA [92,109]. Our data indicate that ZAP binding to L1 RNA is critical for L1 restriction. We demonstrated that ORF1p-FLAG and ZAP interact via an RNA bridge (Fig 1D). Moreover, we showed that overexpression of the ZAP zinc-finger domain more potently inhibits L1 retrotransposition than overexpression of wild type ZAP-L or ZAP-S (Fig 3A), and that the ZAP-S zinc-finger domain is required to inhibit L1 retrotransposition (Fig 3A and S4A Fig). In addition to our genetic and biochemical data, fluorescence microscopy revealed that: 1) co-transfected ZAP-S, L1 RNA, and ORF1p co-localize in the cytoplasm of HeLa cells; 2) the ZAP-S zinc-finger domain is necessary and sufficient for the co-localization of ZAP-S, L1 RNA, and ORF1p; 3) endogenous ZAP co-localizes with transfected ORF1p in HeLa cells; and 4) endogenous ORF1p interacts with transfected ZAP-S in human PA-1 embryonic carcinoma cells (Figs 5A–5E and 6A–6D). Thus, the data suggest that ZAP interacts with L1 RNA in order to mediate L1 restriction.

Notably, the zebrafish ZfL2-2 retrotransposon lacks a homolog to ORF1 and only encodes a single ORF that contains an apurinic/apyrimidinic endonuclease-like (EN) and a reverse transcriptase (RT) domain [95]. The finding that ZAP-S efficiently restricts ZfL2-2 retrotransposition further indicates that ZAP-S likely restricts retrotransposition by interacting with LINE RNA. Although a ZAP consensus RNA target sequence/motif has not yet been identified, evidence suggests that ZAP recognizes long RNA stretches (>500 nucleotides) and/or specific RNA tertiary structure [91,92]. The ability of ZAP to inhibit non-human LINE elements suggests that ZAP may not recognize a particular LINE linear consensus RNA sequence, but instead may recognize an unidentified structural feature common to certain LINE RNAs [91,92].

Evidence suggests that ZAP prevents the accumulation of viral RNAs in the cytoplasm [72]. ZAP-S overexpression significantly reduced the amount of polyadenylated, full-length L1 RNA (Fig 4B), which would be expected to inhibit retrotransposition by limiting the supply of L1 mRNA available for translation and as a template for TPRT. Notably, while ZAP-S selectively inhibited the accumulation of full-length L1 transcripts, it did not dramatically affect the accumulation of shorter, spliced and/or polyadenylated L1 RNAs (Fig 4B) [99–101,110]. Thus, ZAP does not appear to affect L1 transcription *per se*, but instead likely affects the post-transcriptional processing of full-length L1 mRNA. In addition to biochemical data, fluorescence microscopy revealed that L1 RNA was depleted from L1 ORF1p cytoplasmic foci in the presence of ZAP (S5A Fig). The depletion of RNA from L1 cytoplasmic foci was dependent on the ZAP-S zinc-finger domain. Based on these data it is likely that ZAP prevents the accumulation of cytoplasmic L1 mRNA.

Previous studies have shown that ZAP also suppresses the expression of viral proteins [111–113]. Western blot experiments demonstrated that the overexpression of ZAP-S inhibited the accumulation of L1 ORF1p and L1 ORF2p in whole cell lysates and RNPs derived from transfected HeLa cells (Fig 4D and S3B Fig). In agreement with western blot experiments, confocal microscopy experiments showed that tGFP-tagged ZAP-S inhibited the expression of ORF1p in transfected HeLa cells and that the ZAP-S zinc-finger domain is critical for the inhibition of ORF1p expression (S5B Fig). ZAP-S expression also inhibited Alu retrotransposition (Fig 3B), which depends on ORF2p to be supplied *in trans* by L1 [49]. In contrast to these data, ZAP-S overexpression did not significantly affect the expression and/or accumulation of EGFP or other endogenous proteins (*e*.*g*., eiF3 and tubulin) (Fig 4D and S3B Fig). Thus, ZAP may preferentially limit the accumulation of ORF1p and ORF2p by interacting with L1 mRNA.

**Fig 7:**
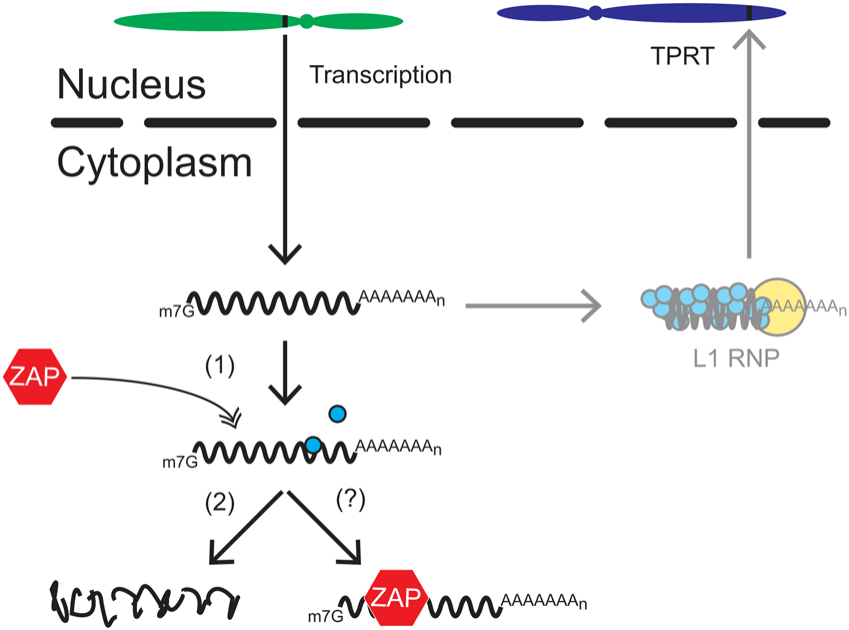
A working model for how ZAP restricts L1 retrotransposition. Once a genomic L1 (black rectangle located on the green chromosome) is transcribed, the resultant bicistronic L1 mRNA is exported to the cytoplasm for translation. L1 ORF1p (blue circles) and ORF2p (yellow circle) bind back to L1 mRNA to form an L1 RNP. The L1 RNP gains access to the nucleus where a new L1 copy is inserted into genomic DNA by the process of TPRT (black rectangle located on the blue chromosome). In ZAP-mediated restriction, ZAP (red hexagon) interacts with L1 mRNA in the cytoplasm (1), which we propose leads to the destabilization of L1 RNA (2) and/or a block in translation (?) through the recruitment of other cellular factors (*e*.*g*., SG associated proteins, RNA decay proteins) involved in RNA metabolism.

In sum, our data suggest that ZAP restricts L1 retrotransposition by preventing the accumulation of cytoplasmic L1 RNA. Notably, a recent study suggests that ZAP may interfere with translation of viral RNA, and that translation inhibition may precede viral RNA destruction [113]. Although a reduction in L1 RNA could explain the observed decrease in L1 protein expression, it also is conceivable that the interaction between ZAP and L1 RNA could interfere with L1 translation (Fig 7).

It remains unclear how ZAP might destabilize full-length L1 RNA to restrict retrotransposition. Evidence suggests that ZAP recruits exosome components [109] along with other proteins involved in RNA degradation [111,114] to destroy viral RNA. Interestingly, immunofluorescence microscopy experiments revealed that ORF1p and ZAP co-localize with components of cytoplasmic SGs (S4B and S4D Fig), which contain numerous RNA binding proteins involved in cytosolic RNA metabolism [reviewed in 115]. Indeed, ZAP previously has been shown to localize to SGs [108] and SGs have been suggested to play a role in regulating L1 retrotransposition [104] and viral pathogenesis [reviewed in 116]. The co-localization of ORF1p and ZAP with SGs also suggests that ZAP may possibly inhibit L1 translation, as SG assembly is stimulated by translational arrest [reviewed in 115]. Thus, we propose that ZAP interacts directly with L1 RNA in the cytoplasm, which likely results in the recruitment in SG components and/or other cellular factors involved in RNA metabolism to destabilize L1 RNA and/or block translation (Fig 7).

ZAP exhibits antiviral activity against a variety of viruses such as MLV [72], alphaviruses [117], filoviruses [118], HIV-1 [111], and hepatitis-B virus [119]. Interestingly, many putative L1 restriction factors also are involved in antiviral defense (*i*.*e*., a subset of APOBEC3 proteins, TREX1, MOV10, SAMHD1, and RNaseL). L1 elements have been active in mammalian genomes for ~160 million years [120–122]. Thus, it is tempting to speculate that some host factors, such as ZAP, may have first evolved to combat endogenous retrotransposons and subsequently were co-opted as viral restriction factors [90,123–125]. Indeed, identifying host factors that modulate L1 retrotransposition may prove to be an effective strategy to identify host antiviral factors.

## Methods

### Cell culture

HeLa-JVM cells were grown in high-glucose DMEM (Gibco) supplemented with 10% FBS (Gibco), 100 U/mL penicillin-streptomycin (Invitrogen), and 0.29 mg/mL L-glutamine (Gibco) [30]. HeLa-HA [126] and PA-1 [107] cells were grown in MEM (Gibco) with 10% FBS, 100 U/mL penicillin-streptomycin, 0.29 mg/mL L-glutamine, and 0.1 mM nonessential amino acids (Gibco). Cell lines were maintained at 37°C with 7% CO_2_ in humidified incubators (Thermo Scientific).

### Plasmids

Oligonucleotide sequences and cloning strategies used in this study are available upon request. All human L1 plasmids contain the indicated fragments of L1.3 (accession no. L19088) [5] DNA cloned into pCEP4 (Invitrogen) unless otherwise indicated. A CMV promoter augments expression of all L1 and cDNA expressing plasmids unless noted otherwise. L1 plasmids also contain an SV40 polyadenylation signal that is located downstream of the native L1 polyadenylation signal. All plasmid DNA was prepared with a Midiprep Plasmid DNA Kit (Qiagen).

The following cDNA expression plasmids were obtained from OriGene: CDK9 (SC119344); DDX21 (SC108813); GNB2L1 (SC116322); hnRNPDL (SC107613); MOV10 (SC126015); MATR3 (SC113375); ZAP-S (ZC3HAV1 transcript variant 2) (SC101064); ZAP-S-tGFP (GFP-tagged ZC3HAV1 transcript variant 2) (RG208070); hnRNPA2B1 (SC313092); IGF2BP3 (SC111161); PURA (SC127792); UPF1 (SC118343).

The following cDNA expression plasmids were obtained from Open Biosystems: hnRNPL (6174088); LARP2 (5164712); LARP4 (5219803); SYNCRIP (5495201).

The following cDNA expression plasmids were obtained from Addgene: rZAP (pcDNA4-TO-Myc-rZAP; Addgene plasmid#: 17381, kindly provided by Dr. Stephen Goff) (Gao et al., 2002) and ZAP-L (pcDNA4 huZAP(L); Addgene plasmid#: 45907, kindly provided by Dr. Harmit Malik) [90].

pJM101/L1.3: is a pCEP4-based plasmid that expresses a human L1 (L1.3) equipped with an *mneoI* retrotransposition indicator cassette. L1 expression is augmented by a CMV promoter located upstream of the L1 5' UTR and an SV40 polyadenylation signal that is located downstream of the native L1 polyadenylation signal [5,30,127,128]

pJM101/L1.3FLAG: was derived from pJM101/L1.3 and contains a single FLAG epitope on the carboxyl-terminus of ORF1p. Dr. Huira Kopera (University of Michigan Medical School) constructed the plasmid.

pAlu*neo* ^Tet^ : expresses an Alu element cloned from intron 5 of the human *NF1* gene [129] that is marked with the *neo* ^Tet^ reporter gene. The reporter [130] was subcloned upstream of the Alu poly adenosine tract [49].

pCEP/GFP: is a pCEP4 based plasmid that expresses the humanized renilla green fluorescent protein (hrGFP) coding sequence from phrGFP-C (Stratagene), which is located downstream of the pCEP4 CMV promoter [33].

pJJ101/L1.3: is a pCEP4 based plasmid that contains an active human L1 (L1.3) equipped with an *mblastI* retrotransposition indicator cassette [85].

pJJ105/L1.3: is similar to pJJ101/L1.3, but contains a D702A missense mutation in the RT active site of L1.3 ORF2 [85].

pJM101/L1.3Δneo: is a pCEP4 based plasmid that contains an active human L1 (L1.3) [35].

pLRE3-EF1-*mEGFP*ΔIntron: is a pBSKS-II+ based plasmid that expresses an active human L1 (LRE3) that is tagged with an *EGFP* cassette (*mEGFPI*) containing an antisense, intronless copy of the EGFP gene. A UbC promoter drives EGFP expression. An EF1α promoter drives L1 expression [103].

pAD2TE1: is similar to pJM101/L1.3 except that it was modified to contain a *T7 gene10* epitope-tag on the carboxyl-terminus of ORF1p and a TAP epitope-tag on the carboxyl-terminus of ORF2p. The 3′-UTR contains the *mneoI* retrotransposition indicator cassette [26].

pJBM2TE1: is similar to pAD2TE1 except that the pCEP4 backbone was modified to contain the puromycin resistance (PURO) gene in place of the hygromycin resistance gene.

pLRE3-*mEGFPI*: is a pCEP4 based plasmid that contains an active human L1 (LRE3) equipped with an *mEGFPI* retrotransposition indicator cassette [97,106]. The pCEP4 backbone was modified to contain a puromycin resistance (PURO) gene in place of the hygromycin resistance gene. The CMV promoter also was deleted from the vector; thus, L1 expression is driven only by the native 5′ UTR [97].

pJM111-LRE3-*mEGFPI*: is identical to pLRE3-*mEGFPI* except that it contains two missense mutations in ORF1 (RR261-262AA), which render the L1 retrotransposition-defective [30]. Mr. William Giblin (University of Michigan Medical School) constructed the plasmid [69].

pG_F_ 21: contains an 8.8 kb fragment which includes a full length mouse G_F_21 L1 element that contains the *mneoI* indicator cassette [94].

pZfL2-2: is a pCEP4 based plasmid that contains the ZfL2-2 ORF (ZL15, accession no. AB211150) cloned upstream of the *mneoI* indicator cassette [95].

pCEP4smL1: contains a codon optimized full-length mouse element (derived from L1_spa_) containing the *mneoI* indicator cassette [96].

ZAP-S/1-311: encodes the ZAP-S amino acid sequence from 1–311 and the following sequence of non-templated amino acids (IIIYTGFLFCCGFFFFFFFLEGVSLCCPGWS).

ZAP-S/Δ72–372: was derived by deleting the *Sfo*I*-Xho*I fragment from ZC3HAV1 transcript variant 2 (OriGene, SC101064), and expresses a ZAP-S mutant protein that lacks amino acid sequence from 72–372.

ZAP-S/Δ310-645-tGFP: expresses a ZAP-S mutant protein that lacks amino acid sequence from 310–645 and contains a carboxyl terminus tGFP epitope tag.

ZAP-S/Δ72-372-tGFP: expresses a ZAP-S mutant protein that lacks amino acid sequence from 72–372 and contains a carboxyl terminus tGFP epitope tag.

LARP5: was derived by cloning LARP5 cDNA (Open Biosystems, 40118844) into pcDNA3 (Invitrogen).

LARP1: was constructed by cloning the LARP1 cDNA (Open Biosystems, 3138935) into pcDNA3 (Invitrogen).

pK_A3A: expresses HA-tagged APOBEC3A and was a generous gift from Dr. Brian Cullen [131].

pDCP1α-GFP: expresses a GFP-tagged version of DCP1α and was a generous gift from Dr. Gregory Hannon [132].

pG3BP-GFP: expresses a GFP-tagged version of G3BP and was a generous gift from Dr. Jamal Tazi [133].

pcDNA6/TR: expresses the blasticidin resistance gene and was obtained from Invitrogen.

### Immunoprecipitation of L1 ORF1p

HeLa-JVM cells were seeded in T-175 flasks (BD Falcon) at ~6–8×10^6^ cells/flask and transfected the next day with 20 μg of plasmid DNA using 60 μL of FuGENE HD (Promega). Approximately 48 hours post-transfection, hygromycin B (Gibco) (200 μg/mL) was added to the medium to select for transfected cells. After approximately one week of hygromycin selection, cells were washed 3 times with ice cold PBS and collected with a rubber policeman into 50 mL conical tubes (BD Falcon). Cells were then pelleted at 1,000×g and frozen at -80°C. To produce whole cell lysates (WCL), frozen cell pellets were rapidly thawed and then lysed in ~3 mL (1 mL lysis buffer per 100 mg of cell pellet) of lysis buffer (20 mM Tris-HCl (pH 7.5), 150 mM NaCl, 10% glycerol, 1 mM EDTA, 0.1% IGEPAL CA-630 (Sigma), 1X complete EDTA-free protease inhibitor cocktail (Roche)) on ice for 30 minutes. WCLs were then centrifuged at 15,000×g for 15 minutes at 4°C. Supernatants were transferred to a clean tube and protein concentration was determined using the Bradford reagent assay (BioRad). For the IP, ~1 mL of the supernatant (~3 mg total protein) was pre-cleared with ~15 μL (packed gel volume) of mouse IgG-agarose beads (Sigma) for 4 hours at 4°C. Pre-cleared supernatants were then mixed with ~15 μL (packed gel volume) of EZview Red ANTI-FLAG M2 Affinity Gel (Sigma) and incubated overnight with rotation at 4°C. The beads then were rinsed 3x with 0.5 mL of lysis buffer, and then washed 3 times with 0.5 mL of lysis buffer for 10 minutes per wash on ice with gentle agitation. Protein complexes were eluted from the beads by adding ~70 μL of 2X NuPAGE LDS Sample Buffer (Novex), supplemented with NuPAGE Sample Reducing Agent (Novex), directly to the washed beads and incubating for 10 minutes at 70°C. Following incubation, the beads were pelleted and the sample was transferred to a fresh tube. For SDS-PAGE analysis, 20 μL of the IP were loaded onto a 4–15% gradient midi-gel (BioRad) and run under reducing conditions. Gels were silver stained using the SilverQuest Silver Staining Kit (Novex) to visualize proteins.

### Protein identification by LC-MS/MS

The Proteomics Facility at the Fred Hutchinson Cancer Research Center (Seattle, WA) conducted protein identification experiments. Excised silver-stained gel slices were destained and subjected to in-gel proteolytic digestion with trypsin as described [134]. Following gel-slice digestion, the digestion products were desalted using C18-micro ZipTips (Millipore) and were dried by vacuum centrifugation. The resultant peptide samples were resuspended in 7 μL of 0.1% formic acid and 5 μL were analyzed by liquid chromatography coupled to tandem mass spectrometry (LC-MS/MS). LC-MS/MS analysis was performed using an LTQ Orbitrap XL mass spectrometer (Thermo Scientific). The LC system, configured in a vented format [135], consisted of a fused-silica nanospray needle (PicoTip emitter, 50 μm ID) (New Objective) packed in-house with Magic C18 AQ 100A reverse-phase medium (25 cm) (Michrom Bioresources Inc.) and a trap (IntegraFrit Capillary, 100 μm ID) (New Objective) containing Magic C18 AQ 200A reverse-phase medium (2 cm) (Michrom Bioresources Inc.). The peptide samples were loaded onto the column and chromatographic separation was performed using a two mobile-phase solvent system consisting of 0.1% formic acid in water (A) and 0.1% acetic acid in acetonitrile (B) over 60 min from 5% B to 40% B at a flow rate of 400 nL/minutes. The mass spectrometer operated in a data-dependent MS/MS mode over the *m/z* range of 400–1800. For each cycle, the five most abundant ions from each MS scan were selected for MS/MS analysis using 35% normalized collision energy. Selected ions were dynamically excluded for 45 seconds.

For data analysis, raw MS/MS data were submitted to the Computational Proteomics Analysis System (CPAS), a web-based system built on the LabKey Server v11.2 [136] and searched using the X!Tandem search engine [137] against the International Protein Index (IPI) human protein database (v3.75), which included additional common contaminants such as BSA and trypsin. Search results were compared between the pJM101/L1.3FLAG lane and the pJM101/L1.3 lane to generate a list of candidate L1 ORF1p associated proteins unique to the pJM101/L1.3FLAG immunoprecipitation. The search output files were analyzed and validated by ProteinProphet [138]. Peptide hits were filtered with PeptideProphet [139] error rate ≤0.05, and proteins with probability scores of ≥0.95 were accepted. Suspected contaminants (*e*.*g*. keratin) were filtered from the final L1 RNP candidate list.

### L1 retrotransposition assays

The cultured cell retrotransposition assay was carried out essentially as described [30,71]. For retrotransposition assays with L1 constructs tagged with *mblastI*, HeLa-JVM cells were seeded at ~1–2×10^4^ cells/well in a 6-well plate (BD Falcon). Within 24 hours, each well was transfected with 1 μg of plasmid DNA (0.5 μg L1 plasmid + 0.5 μg cDNA plasmid or pCEP4) using 3 μL of FuGENE 6 transfection reagent (Promega). Four days post-transfection, blasticidin (EMD Millipore) containing medium (10 μg/mL) was added to cells to select for retrotransposition events. Medium was changed every two days. After ~8 days of selection, cells were washed with PBS, fixed, and then stained with crystal violet to visualize colonies. To control for transfection efficiency and off-target effects of cDNA plasmids, in parallel with retrotransposition assays, HeLa-JVM cells were plated in 6-well plates at 500–1,000 cells/well and transfected with 0.5 μg pcDNA6/TR (Invitrogen) plasmid + 0.5 μg cDNA plasmid using 3 μL of FuGENE 6 transfection reagent (Promega). The pcDNA6/TR control assays were treated with blasticidin in the same manner as for retrotransposition assays.

For retrotransposition assays with L1 constructs tagged with *mneoI*, HeLa-JVM cells were transfected as described above. Two days after transfection, cells were treated with medium supplemented with G418 (Gibco) (500 μg/mL) for ~10–12 days. As a control, HeLa cells were plated at ~2×10^4^ cells/well in a 6-well plate and transfected with 0.5 μg pcDNA3 (Invitrogen) plasmid + 0.5 μg cDNA plasmid using 3 μL of FuGENE 6 transfection reagent (Promega). The pcDNA3 control assays were treated with G418 in the same manner as for retrotransposition assays.

### Alu retrotransposition assays

For Alu retrotransposition assays [49], ~4×10^5^ HeLa-HA cells were plated per well of a 6-well plate (BD Falcon) and transfected with 0.67 μg of pJM101/L1.3Δneo + 0.67 μg of pAlu*neo* ^Tet^ + 0.67 μg of cDNA plasmid using 6 μL FuGENE HD (Promega). Three days post-transfection, cells were grown in the presence of G418 (500μg/mL) to select for Alu retrotransposition events. As a control, HeLa-HA cells were plated at ~4×10^5^ cells/well in a 6-well plate and transfected with 0.67 μg of pcDNA3 (Invitrogen) + 0.67 μg of pAlu*neo* ^Tet^ + 0.67 μg of cDNA plasmid using 6 μL of FuGENE HD (Promega). The pcDNA3 control assays were treated with G418 in the same manner as for Alu retrotransposition assays.

### siRNA knockdown and pLRE3-*mEGFPI* retrotransposition assays

In experiments to study the effect of endogenous proteins on L1 retrotransposition, HeLa cells (~8×10^5^ cells) were plated in 60 mm tissue culture dishes (BD Falcon). The next day, the cells were transfected with 50 nM of a control siRNA pool (D-001810-10, ON-TARGETplus Non-targeting Pool, Thermo Scientific) or siRNA against ZAP (L-017449-01-0005, ON-TARGETplus Human ZC3HAV1 (56829) siRNA—SMARTpool, Thermo Scientific) or MOV10 (L-014162-00-0005, ON-TARGETplus Human MOV10 (4343) siRNA—SMARTpool, Thermo Scientific) using the DharmaFECT 1 transfection reagent (Thermo Scientific). Twenty-four hours after siRNA treatment, cells were transfected with pLRE3-*mEGFPI* or pJM111-LRE3-*mEGFPI* (5 μg), using 15 μL of FuGENE HD transfection reagent (Roche). After 48 hours, cells were trypsinized and an aliquot of the cells (~2×10^6^ cells) was used to monitor endogenous protein levels (72 hours after siRNA treatment) by western blot analysis (see below for list of primary antibodies). Blots were analyzed using an Odyssey CLx (LI-COR) with the following secondary antibodies: IRDye 800CW Donkey anti-Rabbit IgG (1:10,000) (LI-COR) and IRDye 680RD Donkey anti-Mouse IgG (1:10,000) (LI-COR). Knockdown efficiencies were calculated using LI-COR Image Studio Software (v3.1.4) and are the average of three independent experiments. Endogenous tubulin was used as the normalization control. The remaining cells were re-plated at ~2×10^5^ cells/well of a 6-well plate and cultured in medium supplemented with puromycin (5 μg/ml, Gibco/Life Technologies) to select for cells transfected with pLRE3-*mEGFPI*. After 4 days of puromycin selection, the percentage of GFP positive cells was determined by flow cytometry using an Accuri C6 flow cytometer (BD Biosciences).

### RNP isolation

RNPs were isolated as previously described [37]. Briefly, HeLa-JVM cells were seeded onto 60 mm tissue culture dishes (BD Falcon) and 24 hours later cells were co-transfected with 2.5 μg of pJBM2TE1 and 2.5 μg of the indicated cDNA plasmid using 15 μL of FuGENE HD (Promega). Approximately two days after transfection, puromycin (5 μg/mL) was added to culture medium to select for cells transfected with pJBM2TE1. After ~3 days of puromycin selection (5 days after transfection), cells were lysed in RNP lysis buffer (150 mM NaCl, 5 mM MgCl_2_, 20 mM Tris-HCl (pH 7.5), 10% glycerol, 1mM DTT, 0.1% NP-40, and 1x complete EDTA-free protease inhibitor cocktail (Roche)). Following lysis, whole cell lysates were centrifuged at 12,000xg for 10 minutes at 4°C, and then the cleared lysate was layered onto a sucrose cushion (8.5% and 17% sucrose) and subjected to ultracentrifugation at 4°C for 2 hours at 178,000xg. The supernatant was discarded and the resulting pellet was resuspended in water supplemented with 1x complete EDTA-free protease inhibitor cocktail (Roche). Approximately 20 μg (total protein) of the RNP sample or ~30 μg (total protein) of the cleared whole cell lysate (supernatant post 12,000xg centrifugation) were then analyzed by western blot. Blots were analyzed using an Odyssey® CLx (LI-COR) with the following secondary antibodies: IRDye 800CW Donkey anti-Rabbit IgG (1:10,000) (LI-COR) and IRDye 680RD Donkey anti-Mouse IgG (1:10,000) (LI-COR).

To simultaneously analyze the effects of ZAP-S on ORF1p and EGFP protein expression, HeLa-JVM cells were seeded onto 10 cm dishes (~2.7×10^6^ cells/dish) (BD Falcon) and transfected with 10 μg of plasmid DNA (5.0 μg pLRE3-EF1A-*mEGFP*ΔIntron + 5.0 μg cDNA plasmid or pCEP4) using 30 μL of FuGENE HD. After 48 hours, cells were harvested with trypsin and then subjected to flow cytometry to isolate GFP expressing cells. Approximately 1.2–1.7×10^6^ GFP positive cells were collected for each transfection condition using a MoFlo Astrios cell sorter (Beckman Coulter). The GFP gate was set using untransfected HeLa-JVM cells. The sorted cells were lysed as described in the IP procedure and lysates were then subjected to western blotting using standard procedures. For all other protein expression analyses, HeLa-JVM cells were seeded at ~4×10^5^ cells/well in 6-well plates and transfected with 2 μg of plasmid DNA with 6 μL of FuGENE HD. Cells were collected 48 hours after transfection using a rubber policeman and lysates were prepared as described above. Western blots were visualized using either the SuperSignal West Femto Chemiluminescent Substrate (Pierce) or SuperSignal West Pico Chemiluminescent Substrate (Pierce) and Hyperfilm ECL (GE Healthcare).

### Northern blots

HeLa-JVM cells were seeded in T-175 flasks (BD Falcon) and transfected with 20 μg of plasmid DNA (10 μg pJM101/L.13Δneo + 10 μg cDNA plasmid) using 60 μL FuGENE HD. Two days after transfection, cell pellets were collected and frozen at -80°C. Frozen cell pellets were then thawed and total RNA was extracted with TRIzol reagent (Ambion), and then poly(A)+ RNA was prepared from total RNA using an Oligotex mRNA kit (Qiagen). Each sample (~1.5 μg of poly(A)+ RNA) was subjected to glyoxal gel electrophoresis and northern blotting using the NorthernMax-Gly Kit (Ambion) according to the manufacturer’s protocol. Following electrophoresis, RNA was transferred to BrightStar Nylon membranes (Invitrogen) and then cross-linked using UV light. For northern blot detection, membranes were prehybridized for ~ 4 hours at 68°C in NorthernMax Prehybridization/Hybridization Buffer (Ambion), and then incubated with a strand specific RNA probe (final concentration of probe ~ 3×10^6^ cpm ml^-1^) overnight at 68°C. For band quantification, northern blot films were analyzed using ImageJ software [140].

Strand-specific RNA probes were generated using the MAXIscript T3 system (Invitrogen). The 5UTR99 [100] probe corresponds to bases 7–99 of the L1.3 5' UTR and the ORF2_5804 probe corresponds to nucleotides 5560–5804 of the L1.3 sequence. RNA probe templates for T3 reactions were generated by PCR using pJM101/L1.3Δneo as a PCR template with the following primer pairs:

- (5UTR99: 5'-GGAGCCAAGATGGCCGAATAGGAACAGCT-3' and 5'-AATTAACCCTCAAAGGGACCTCAGATGGAAATGCAG-3');
- (ORF2_5804: 5'- GACACATGCACACGTATGTTTATT-3' and 5'- AATTAACCCTCACTAAAGGGTGAGTGAGAATATGCGGTGTTT-3').

The T3 promoter sequence (underlined) was added to the reverse primer of each primer pair. The pTRI-β-actin-125-Human Antisense Control Template (Applied Biosystems) was used in T3 reactions as a template to generate the β-actin RNA probe. Each northern blot experiment was independently repeated three times with similar results.

### Immunofluorescence microscopy

Immunofluorescence microscopy was performed essentially as described [26] with modifications. Briefly, cells were plated on round glass cover slips (Fisher) in a 12-well plate or into 4-well chambered glass slides (Fisher) and transfected ~24 hours later with 0.5 μg of plasmid DNA using 1.5 μL of FuGENE 6 transfection reagent. To visualize proteins, approximately 48 hours post-transfection cells were washed with 1x PBS, fixed with 4% paraformaldehyde for 10 minutes and then treated with ice-cold methanol for 1 minute. Next, cells were incubated for 30 minutes at 37°C in 1x PBS + 3% BSA. Cells then were incubated with primary antibodies in 1x PBS + 3% BSA for 1 hour at 37°C. Cells were washed three times with 1x PBS (10 minutes per wash) and then incubated with appropriate, fluorescently-labeled secondary antibodies diluted in 1x PBS for 30 minutes at 37°C. The following secondary antibodies were used for indirect immunofluorescence: Alexa Fluor 488 conjugated Goat anti-Mouse and Goat anti-Rabbit (Invitrogen) (1:1000), Alexa Fluor 546 conjugated Goat anti-Mouse and Goat anti-Rabbit IgG (Invitrogen) (1:1000), and Cy5 conjugated Donkey anti-Rabbit IgG (H+L) (Jackson ImmunoResearch) (1:100). To obtain images, a cover slip and/or slide was visually scanned and representative images were captured using a Leica SP5X confocal microscope (63x/1.4 objective; section thickness 1 μm).

### Combined RNA FISH (fluorescence *in situ* hybridization)/immunofluorescence

Cells were plated on round glass cover slips (Fisher) in a 12-well plate and transfected ~24 hours later with 0.5 μg of plasmid DNA using 1.5 μL of FuGENE 6 transfection reagent. Approximately 48 hours after transfection, cells were fixed with 4% paraformaldehyde for 10 minutes and then permeabilized with 0.2% Triton X-100 in 1x PBS for 7 minutes. Following permeabilization, coverslips were incubated for 5 minutes in FISH (fluorescence *in situ* hybridization) wash buffer (2x SSC, 10% formamide) for 5 minutes. To visualize L1 RNA, coverslips were then incubated with 300 nM FISH probes (sequences below) in FISH hybridization buffer (2x SSC, 10% formamide, 1% dextran sulphate) for ~4 hours at 37°C. Following hybridization, cells were incubated for 30 minutes in FISH wash buffer at 37°C and then incubated with FISH wash buffer + 3% BSA for an additional 30 minutes at 37°C. To visualize L1 ORF1p by immunofluorescence, coverslips then were incubated with αORF1p antibodies (1:2000) in 1x PBS + 3% BSA for 1 hour at 37°C. Cells were washed three times with 1x PBS (10 minutes per wash). Cells were incubated with Alexa Fluor 546 conjugated Goat anti-Rabbit IgG (Invitrogen) (1:1000) in 1x PBS + DAPI (50 ng/mL) for 30 minutes at 37°C. Coverslips were mounted on slides with VECTASHIELD mounting media (Vector Laboratories). Combined RNA FISH/immunofluorescence samples were imaged with a Zeiss Axioplan2 microscope (63x objective; Axiovision 4.8 software). RNA FISH/immunofluorescence images (Fig 6A–6D) were globally processed using the Photoshop CS6 (version 13.0 x64) Levels tool to adjust input levels. The L1 RNA was labeled using 21 Quasar670-labelled anti-sense oligonucleotide probes complimentary to sequences within the L1.3 5' UTR (probes were designed and produced by Biosearch Technologies, Petaluma, CA). The sequences of the 21 L1 probes are as follows: 5'-aaatcaccgtcttctgcgtc-3', 5'-ggtacctcagatggaaatgc-3', 5'-cactccctagtgagatgaac-3', 5'-ccctttctttgactcagaaa-3', 5'-aatattcgggtgggagtgac-3', 5'-cttaagccggtctgaaaagc-3', 5'-caggtgtgggatatagtctc-3', 5'-tgctagcaatcagcgagatt-3', 5'-ttgcagtttgatctcagact-3', 5'-tttgtttacctaagcaagcc-3', 5'-cagaggtggagcctacagag-3', 5'-ctgtctttttgtttgtctgt-3', 5'-cacttaagtctgcagaggtt-3', 5'-ctctcttcaaagctgtcaga-3', 5'-ttgaggaggcagtctgtctg-3', 5'-ctgcaggtctgttggaatac-3', 5'-ttctaacagacaggaccctc-3', 5'-cctttctggttgttagtttt-3', 5'-gatgggttttcggtgtagat-3', 5'-gtctttgatgatggtgatgt-3', 5'-tttgtggttttatctacttt-3'.

### Primary antibodies

Polyclonal antibodies against peptide sequences 31–49 of L1.3 ORF1p (αORF1p) were raised in rabbits and affinity-purified (Open Biosystems). αCDK9 (2316), αUPF1 (9435), and αGFP (2955) were obtained from Cell Signaling Technology. αhnRNPL (NBP1-67852), αILF3 (EPR3627), αLARP1 (NBP1-19128), αMATR3 (NB100-1761), αNCL (NB100-1920SS), and αDHX9 (NB110-40579) were obtained from Novus Biologicals. αFAM120A (ab83909), αPURA (ab79936), and αHA tag (ab9110) were obtained from Abcam. αMOV10 (SAB1100141), αZAP (Anti-ZC3HAV1 (HPA047818)), and αTubulin (T9026) were obtained from Sigma. αZC3HAV1 (16820-1-AP) was obtained from Proteintech. αeIF3 (p110) (sc-28858) was obtained from Santa Cruz Biotechnology. αT7-Tag mouse monoclonal (69522–3) was obtained from Novagen. αTAP rabbit polyclonal (CAB1001) was obtained from Thermo Scientific.

## Supporting Information

S1 Fig Supporting data for Fig 1. *(A) The FLAG epitope on ORF1p is compatible with retrotransposition*: Constructs were tested in a transient HeLa cell retrotransposition assay [30,71]. The X-axis indicates the L1 plasmid. The Y-axis indicates the retrotransposition efficiency. Retrotransposition assays were normalized to pJM101/L1.3 (100%). The pJM105/L1.3 plasmid serves as a negative control and harbors a point mutation in the ORF2p RT domain that renders the element inactive [30]. Representative results from a single experiment are depicted below the graph. The assay was repeated two times with similar results. *(B) Immunoprecipitation reactions conducted using various wash conditions*: Top panel: HeLa cells were transfected with pCEP4, pJM101/L1.3, or pJM101/L1.3FLAG and were subjected to lysis using two different salt concentrations (500 mM NaCl (left gel) or 150 mM NaCl (right gel)). Shown are the images of silver stained gels from immunoprecipitation reactions. The black rectangles indicate the cropped image depicted in Fig 1B. Molecular weight standards (~kDa) are shown on the left side of the gel. Bottom panel: Image of full western blot used in Fig 1C. The black rectangle indicates the cropped lanes depicted in Fig 1C. Molecular weight standards (~kDa) are shown on the left side of the gel. *(C) Immunoprecipitation reactions under different lysis buffer conditions*: Silver stained gels of IP fractions from untransfected HeLa (HeLa UTF) or HeLa cells transfected with pJM101/L1.3FLAG. Lysis buffer contained either 0.1% CHAPS (left gel) or 1.0% Triton X-100 (right gel). Black arrows correspond to the approximate location of ORF1p-FLAG; black bars indicate the approximate location of proteins enriched in the pJM101/L1.3FLAG lane. Molecular weight standards (kDa) are shown on the left side of the gels.
(TIF)

S2 Fig Supporting data for Fig 3. *(A-C) Transfected ZAP is expressed in HeLa cells*: Western blots of whole cell lysates demonstrate the expression of ZAP-L (panel A) and ZAP-S and ZAP-S/∆72–372 (panels B and C) 48 hours post-transfection. UTF indicates untransfected HeLa cells. The antibodies are indicated at the right side of the blots. Blue arrows indicate the approximate locations of the ZAP proteins. Tubulin serves as a loading control. Molecular weight standards (kDa) are shown on the left side of the blots. *(D) The depletion of endogenous ZAP enhances L1 retrotransposition*: Flow cytometry was used to determine the percentage of EGFP-positive, live-gated cells for each siRNA transfection condition (noted above the plots). The X-axis depicts the scattering at 533 nm; the Y-axis depicts the scattering at 585 nm. The EGFP-positive gate was set using the retrotransposition-deficient negative control, pJM111-LRE3-*mEGFPI* [30,69].
(TIF)

S3 Fig Supporting data for Fig 4. ZAP-S preferentially suppresses the expression of ORF1p. *(A) A schematic of the pLRE3-EF1-mEGFPΔIntron*: pLRE3-EF1-*mEGFP*ΔIntron expresses a human L1 (LRE3) that is tagged with an *mEGFPI* expression cassette that lacks an intron. The human elongation factor-1 alpha (EF1α) promoter (arrow) augments L1 transcription. The ubiquitin C (UbC) promoter (upside down arrow) drives EGFP transcription. *(B) ZAP-S inhibits ORF1p expression*: Western blots were conducted using whole cell lysates derived from cells co-transfected with pLRE3-EF1-*mEGFP*ΔIntron and the ZAP-S expression plasmid or pCEP4 indicated above each lane. UTF indicates whole cell lysates from untransfected HeLa cell. Antibodies are indicated on the right side of each blot. Tubulin is used as a loading control. Western blot images depict a representative experiment that was repeated three times with similar results. Notably, upon extended exposure times ORF1p was able to be visualized in the ZAP-S lane. *(C) ZAP-S does not inhibit EFGP expression and/or accumulation*: HeLa cells were co-transfected with pLRE3-EF1-*mEGFP*ΔIntron and the indicated expression plasmids (noted above the plots). Flow cytometry was used to determine the percentage of EGFP-positive, live-gated cells for each condition. UTF indicates untransfected HeLa cells. The EGFP-positive gate was set using the UTF sample as a negative control. The X-axis depicts the percentage of EGFP positive cells. The Y-axis indicates the side scattering profile (SSC). Approximately 1.2–1.7 x 10^6^ GFP positive cells were collected and analyzed for each transfection condition.
(TIF)

S4 Fig Supporting data for Fig 5. *(A) ZAP-S-tGFP inhibits retrotransposition in HeLa cells*: Top panel: Schematics of tGFP-tagged ZAP constructs. Depicted are the relative positions of the zinc-finger domains (light gray rectangles), cysteine-histidine (CCCH) zinc-fingers (vertical black bars), and tGFP tag (green rectangles) ZAP-S expression constructs. Bottom panel: Results of pJJ101/L1.3 retrotransposition assays. The X-axis indicates the cDNA co-transfected with pJJ101/L1.3 or pcDNA6/TR. The Y-axis indicates pJJ101/L1.3 retrotransposition activity (black bars). All values have been normalized to the pCEP4 empty vector control (100%). The numbers above the bar graphs indicate the number of biological replicates performed with each cDNA expression construct. Error bars represent standard deviations. *(B-E) ORF1p and ZAP co-localize with stress granules in HeLa cells*: HeLa cells were co-transfected with pJM101/L1.3Δneo and ZAP-S-tGFP; proteins were visualized by direct immunofluorescence. ORF1p co-localizes with ectopic ZAP-S-tGFP and eIF3 in cytoplasmic foci (panel B). ORF1p co-localizes with ectopic ZAP-S-tGFP (panels A and B), but not with tubulin (panel C). GFP-tagged G3BP co-localizes with endogenous ZAP in cytoplasmic foci (panel D). GFP-tagged DCP1α forms cytoplasmic punctate structures, which do not appear to co-localize with endogenous ZAP (panel E). The right-most image in each panel represents a merged image. The cell type is indicated at the top left (yellow), the protein name is listed on the bottom left, and the name of the primary antibody used (*italicized*) is annotated at the bottom right. Nuclei were stained with DAPI (blue) and the scale bar represents 25 μM.
(TIF)

S5 Fig Supporting data for Fig 6. *(A)* Fluorescence microscopy was used to determine the percentage of cells that contained L1 RNA in cytoplasmic ORF1p foci. The X-axis indicates the plasmid that was co-transfected with pJM101/L1.3. The Y-axis of the graph depicts the percentage of cells where L1 RNA was detected in cytoplasmic ORF1p foci. Experiments were repeated three times. A total of ~60 visual fields (~1600 cells) were examined amongst all three experiments and ~33–41 ORF1p foci containing cells were evaluated for each experimental condition. Error bars represent standard deviations. *(B)* Confocal microscopy was used to determine the number of ORF1p-expressing cells ~48 hours post transfection. The X-axis indicates the plasmid that was co-transfected with pJM101/L1.3Δneo. The Y-axis of the graph depicts the percentage of cells that express ORF1p. Experiments were repeated twice. Each experiment contained two biological replicates and ~1100–1500 cells were enumerated amongst all experiments for each condition. Error bars indicate standard deviations.
(TIF)

S1 Table L1 ORF1p-interacting protein candidates identified by LC-MS/MS. ORF1p-interacting proteins were selected based on the criteria that the protein was unique to the pJM101/L1.3FLAG IP and was identified by two or more unique peptides (peptide error ≤0.05; protein probability ≥0.95). Column 1 = protein name. Column 2 = protein mass. Column 3 = total number of identified peptides. Column 4 = number of unique peptides. Column 5 = the percentage of amino acid coverage for each of the respective proteins. Columns 6–8 = whether the proteins were identified in the indicated studies [47,67,89]. Green highlighting indicates ORF1p-interacting candidates that were verified by western blot (Fig 1D). "Y" in columns 6–8 indicate that the protein was identified as a significant L1-interacting protein by statistical and/or direct biochemical methods; “n.s.” in column 7 indicates that the protein was identified in the study, but that it did not reach the significance threshold set by the authors.
(TIF)

## References

[1] Lander ES, Linton LM, Birren B, Nusbaum C, Zody MC, et al (2001) Initial sequencing and analysis of the human genome. Nature 409: 860–921. 11237011 10.1038/35057062

[2] Beck CR, Garcia-Perez JL, Badge RM, Moran JV (2011) LINE-1 elements in structural variation and disease. Annu Rev Genomics Hum Genet 12: 187–215. 10.1146/annurev-genom-082509-141802 21801021 PMC4124830

[3] Grimaldi G, Skowronski J, Singer MF (1984) Defining the beginning and end of KpnI family segments. Embo J 3: 1753–1759. 6090124 10.1002/j.1460-2075.1984.tb02042.x PMC557592

[4] Brouha B, Schustak J, Badge RM, Lutz-Prigge S, Farley AH, et al (2003) Hot L1s account for the bulk of retrotransposition in the human population. Proc Natl Acad Sci U S A 100: 5280–5285. 12682288 10.1073/pnas.0831042100 PMC154336

[5] Sassaman DM, Dombroski BA, Moran JV, Kimberland ML, Naas TP, et al (1997) Many human L1 elements are capable of retrotransposition. Nat Genet 16: 37–43. 9140393 10.1038/ng0597-37

[6] Beck CR, Collier P, Macfarlane C, Malig M, Kidd JM, et al (2010) LINE-1 retrotransposition activity in human genomes. Cell 141: 1159–1170. 10.1016/j.cell.2010.05.021 20602998 PMC3013285

[7] Cordaux R, Batzer MA (2009) The impact of retrotransposons on human genome evolution. Nat Rev Genet 10: 691–703. 10.1038/nrg2640 19763152 PMC2884099

[8] Kazazian HH Jr., Wong C, Youssoufian H, Scott AF, Phillips DG, et al (1988) Haemophilia A resulting from de novo insertion of L1 sequences represents a novel mechanism for mutation in man. Nature 332: 164–166. 2831458 10.1038/332164a0

[9] Holmes SE, Dombroski BA, Krebs CM, Boehm CD, Kazazian HH Jr. (1994) A new retrotransposable human L1 element from the LRE2 locus on chromosome 1q produces a chimaeric insertion. Nat Genet 7: 143–148. 7920631 10.1038/ng0694-143

[10] Miki Y, Nishisho I, Horii A, Miyoshi Y, Utsunomiya J, et al (1992) Disruption of the APC Gene by a Retrotransposal Insertion of L1 Sequence in a Colon Cancer. Cancer Research 52: 643–645. 1310068

[11] Shukla R, Upton KR, Munoz-Lopez M, Gerhardt DJ, Fisher ME, et al (2013) Endogenous retrotransposition activates oncogenic pathways in hepatocellular carcinoma. Cell 153: 101–111. 10.1016/j.cell.2013.02.032 23540693 PMC3898742

[12] Hancks DC, Kazazian HH Jr. (2012) Active human retrotransposons: variation and disease. Curr Opin Genet Dev 22: 191–203. 10.1016/j.gde.2012.02.006 22406018 PMC3376660

[13] Swergold GD (1990) Identification, characterization, and cell specificity of a human LINE-1 promoter. Mol Cell Biol 10: 6718–6729. 1701022 10.1128/mcb.10.12.6718-6729.1990 PMC362950

[14] Athanikar JN, Badge RM, Moran JV (2004) A YY1-binding site is required for accurate human LINE-1 transcription initiation. Nucleic Acids Res 32: 3846–3855. 15272086 10.1093/nar/gkh698 PMC506791

[15] Becker KG, Swergold GD, Ozato K, Thayer RE (1993) Binding of the ubiquitous nuclear transcription factor YY1 to a cis regulatory sequence in the human LINE-1 transposable element. Hum Mol Genet 2: 1697–1702. 8268924 10.1093/hmg/2.10.1697

[16] Scott AF, Schmeckpeper BJ, Abdelrazik M, Comey CT, O'Hara B, et al (1987) Origin of the human L1 elements: proposed progenitor genes deduced from a consensus DNA sequence. Genomics 1: 113–125. 3692483 10.1016/0888-7543(87)90003-6 PMC7135745

[17] Dombroski BA, Mathias SL, Nanthakumar E, Scott AF, Kazazian HH Jr. (1991) Isolation of an active human transposable element. Science 254: 1805–1808. 1662412 10.1126/science.1662412

[18] Martin SL (1991) Ribonucleoprotein particles with LINE-1 RNA in mouse embryonal carcinoma cells. Mol Cell Biol 11: 4804–4807. 1715025 10.1128/mcb.11.9.4804-4807.1991 PMC361385

[19] Hohjoh H, Singer MF (1996) Cytoplasmic ribonucleoprotein complexes containing human LINE-1 protein and RNA. Embo J 15: 630–639. 8599946 PMC449981

[20] Hohjoh H, Singer MF (1997) Sequence-specific single-strand RNA binding protein encoded by the human LINE-1 retrotransposon. Embo J 16: 6034–6043. 9312060 10.1093/emboj/16.19.6034 PMC1170233

[21] Holmes SE, Singer MF, Swergold GD (1992) Studies on p40, the leucine zipper motif-containing protein encoded by the first open reading frame of an active human LINE-1 transposable element. J Biol Chem 267: 19765–19768. 1328181

[22] Khazina E, Weichenrieder O (2009) Non-LTR retrotransposons encode noncanonical RRM domains in their first open reading frame. Proceedings of the National Academy of Sciences 106: 731–736. 10.1073/pnas.0809964106 19139409 PMC2630067

[23] Martin SL, Bushman FD (2001) Nucleic acid chaperone activity of the ORF1 protein from the mouse LINE-1 retrotransposon. Mol Cell Biol 21: 467–475. 11134335 10.1128/MCB.21.2.467-475.2001 PMC86601

[24] Ergun S, Buschmann C, Heukeshoven J, Dammann K, Schnieders F, et al (2004) Cell type-specific expression of LINE-1 open reading frames 1 and 2 in fetal and adult human tissues. J Biol Chem 279: 27753–27763. 15056671 10.1074/jbc.M312985200

[25] Goodier JL, Mandal PK, Zhang L, Kazazian HH Jr. (2010) Discrete subcellular partitioning of human retrotransposon RNAs despite a common mechanism of genome insertion. Hum Mol Genet 19: 1712–1725. 10.1093/hmg/ddq048 20147320 PMC2850619

[26] Doucet AJ, Hulme AE, Sahinovic E, Kulpa DA, Moldovan JB, et al (2010) Characterization of LINE-1 ribonucleoprotein particles. PLoS Genet 6: pii: e1001150 10.1371/journal.ppat.1001150 20949108 PMC2951350

[27] Feng Q, Moran JV, Kazazian HH Jr., Boeke JD (1996) Human L1 retrotransposon encodes a conserved endonuclease required for retrotransposition. Cell 87: 905–916. 8945517 10.1016/s0092-8674(00)81997-2

[28] Dombroski BA, Feng Q, Mathias SL, Sassaman DM, Scott AF, et al (1994) An in vivo assay for the reverse transcriptase of human retrotransposon L1 in Saccharomyces cerevisiae. Mol Cell Biol 14: 4485–4492. 7516468 10.1128/mcb.14.7.4485-4492.1994 PMC358820

[29] Mathias SL, Scott AF, Kazazian HH Jr., Boeke JD, Gabriel A (1991) Reverse transcriptase encoded by a human transposable element. Science 254: 1808–1810. 1722352 10.1126/science.1722352

[30] Moran JV, Holmes SE, Naas TP, DeBerardinis RJ, Boeke JD, et al (1996) High frequency retrotransposition in cultured mammalian cells. Cell 87: 917–927. 8945518 10.1016/s0092-8674(00)81998-4

[31] Leibold DM, Swergold GD, Singer MF, Thayer RE, Dombroski BA, et al (1990) Translation of LINE-1 DNA elements in vitro and in human cells. Proc Natl Acad Sci U S A 87: 6990–6994. 1698287 10.1073/pnas.87.18.6990 PMC54668

[32] McMillan JP, Singer MF (1993) Translation of the human LINE-1 element, L1Hs. Proc Natl Acad Sci U S A 90: 11533–11537. 8265584 10.1073/pnas.90.24.11533 PMC48018

[33] Alisch RS, Garcia-Perez JL, Muotri AR, Gage FH, Moran JV (2006) Unconventional translation of mammalian LINE-1 retrotransposons. Genes Dev 20: 210–224. 16418485 10.1101/gad.1380406 PMC1356112

[34] Dmitriev SE, Andreev DE, Terenin IM, Olovnikov IA, Prassolov VS, et al (2007) Efficient translation initiation directed by the 900-nucleotide-long and GC-rich 5' untranslated region of the human retrotransposon LINE-1 mRNA is strictly cap dependent rather than internal ribosome entry site mediated. Mol Cell Biol 27: 4685–4697. 17470553 10.1128/MCB.02138-06 PMC1951496

[35] Wei W, Gilbert N, Ooi SL, Lawler JF, Ostertag EM, et al (2001) Human L1 retrotransposition: cis preference versus trans complementation. Mol Cell Biol 21: 1429–1439. 11158327 10.1128/MCB.21.4.1429-1439.2001 PMC99594

[36] Esnault C, Maestre J, Heidmann T (2000) Human LINE retrotransposons generate processed pseudogenes. Nat Genet 24: 363–367. 10742098 10.1038/74184

[37] Kulpa DA, Moran JV (2005) Ribonucleoprotein particle formation is necessary but not sufficient for LINE-1 retrotransposition. Hum Mol Genet 14: 3237–3248. 16183655 10.1093/hmg/ddi354

[38] Kulpa DA, Moran JV (2006) Cis-preferential LINE-1 reverse transcriptase activity in ribonucleoprotein particles. Nat Struct Mol Biol 13: 655–660. 16783376 10.1038/nsmb1107

[39] Kubo S, Seleme Mdel C, Soifer HS, Perez JL, Moran JV, et al (2006) L1 retrotransposition in nondividing and primary human somatic cells. Proc Natl Acad Sci U S A 103: 8036–8041. 16698926 10.1073/pnas.0601954103 PMC1472425

[40] Shi X, Seluanov A, Gorbunova V (2007) Cell divisions are required for L1 retrotransposition. Mol Cell Biol 27: 1264–1270. 17145770 10.1128/MCB.01888-06 PMC1800731

[41] Xie Y, Mates L, Ivics Z, Izsvak Z, Martin SL, et al (2013) Cell division promotes efficient retrotransposition in a stable L1 reporter cell line. Mob DNA 4: 10 10.1186/1759-8753-4-10 23497436 PMC3607998

[42] Luan DD, Korman MH, Jakubczak JL, Eickbush TH (1993) Reverse transcription of R2Bm RNA is primed by a nick at the chromosomal target site: a mechanism for non-LTR retrotransposition. Cell 72: 595–605. 7679954 10.1016/0092-8674(93)90078-5

[43] Cost GJ, Feng Q, Jacquier A, Boeke JD (2002) Human L1 element target-primed reverse transcription in vitro. Embo J 21: 5899–5910. 12411507 10.1093/emboj/cdf592 PMC131089

[44] Cost GJ, Boeke JD (1998) Targeting of human retrotransposon integration is directed by the specificity of the L1 endonuclease for regions of unusual DNA structure. Biochemistry 37: 18081–18093. 9922177 10.1021/bi981858s

[45] Morrish TA, Gilbert N, Myers JS, Vincent BJ, Stamato TD, et al (2002) DNA repair mediated by endonuclease-independent LINE-1 retrotransposition. Nat Genet 31: 159–165. 12006980 10.1038/ng898

[46] Suzuki J, Yamaguchi K, Kajikawa M, Ichiyanagi K, Adachi N, et al (2009) Genetic evidence that the non-homologous end-joining repair pathway is involved in LINE retrotransposition. PLoS Genet 5: e1000461 10.1371/journal.pgen.1000461 19390601 PMC2666801

[47] Taylor MS, Lacava J, Mita P, Molloy KR, Huang CR, et al (2013) Affinity Proteomics Reveals Human Host Factors Implicated in Discrete Stages of LINE-1 Retrotransposition. Cell 155: 1034–1048. 10.1016/j.cell.2013.10.021 24267889 PMC3904357

[48] Gilbert N, Lutz S, Morrish TA, Moran JV (2005) Multiple fates of l1 retrotransposition intermediates in cultured human cells. Mol Cell Biol 25: 7780–7795. 16107723 10.1128/MCB.25.17.7780-7795.2005 PMC1190285

[49] Dewannieux M, Esnault C, Heidmann T (2003) LINE-mediated retrotransposition of marked Alu sequences. Nat Genet 35: 41–48. 12897783 10.1038/ng1223

[50] Ostertag EM, Goodier JL, Zhang Y, Kazazian HH Jr. (2003) SVA elements are nonautonomous retrotransposons that cause disease in humans. Am J Hum Genet 73: 1444–1451. 14628287 10.1086/380207 PMC1180407

[51] Hancks DC, Goodier JL, Mandal PK, Cheung LE, Kazazian HH Jr. (2011) Retrotransposition of marked SVA elements by human L1s in cultured cells. Hum Mol Genet 20: 3386–3400. 10.1093/hmg/ddr245 21636526 PMC3153304

[52] Raiz J, Damert A, Chira S, Held U, Klawitter S, et al (2012) The non-autonomous retrotransposon SVA is trans-mobilized by the human LINE-1 protein machinery. Nucleic Acids Res 40: 1666–1683. 10.1093/nar/gkr863 22053090 PMC3287187

[53] Buzdin A, Ustyugova S, Gogvadze E, Vinogradova T, Lebedev Y, et al (2002) A new family of chimeric retrotranscripts formed by a full copy of U6 small nuclear RNA fused to the 3' terminus of l1. Genomics 80: 402–406. 12376094 10.1006/geno.2002.6843

[54] Garcia-Perez JL, Doucet AJ, Bucheton A, Moran JV, Gilbert N (2007) Distinct mechanisms for trans-mediated mobilization of cellular RNAs by the LINE-1 reverse transcriptase. Genome Res 17: 602–611. 17416749 10.1101/gr.5870107 PMC1855177

[55] Weber MJ (2006) Mammalian Small Nucleolar RNAs Are Mobile Genetic Elements. PLoS Genet 2: e205 17154719 10.1371/journal.pgen.0020205 PMC1687206

[56] Levin HL, Moran JV (2011) Dynamic interactions between transposable elements and their hosts. Nature reviews Genetics 12: 615–627. 10.1038/nrg3030 21850042 PMC3192332

[57] Yoder JA, Walsh CP, Bestor TH (1997) Cytosine methylation and the ecology of intragenomic parasites. Trends Genet 13: 335–340. 9260521 10.1016/s0168-9525(97)01181-5

[58] Bourc'his D, Bestor TH (2004) Meiotic catastrophe and retrotransposon reactivation in male germ cells lacking Dnmt3L. Nature 431: 96–99. 15318244 10.1038/nature02886

[59] Siomi MC, Sato K, Pezic D, Aravin AA (2011) PIWI-interacting small RNAs: the vanguard of genome defence. Nat Rev Mol Cell Biol 12: 246–258. 10.1038/nrm3089 21427766

[60] Aravin AA, Sachidanandam R, Girard A, Fejes-Toth K, Hannon GJ (2007) Developmentally regulated piRNA clusters implicate MILI in transposon control. Science 316: 744–747. 17446352 10.1126/science.1142612

[61] Richardson SR, Narvaiza I, Planegger RA, Weitzman MD, Moran JV (2014) APOBEC3A deaminates transiently exposed single-strand DNA during LINE-1 retrotransposition. Elife 3: e02008 10.7554/eLife.02008 24843014 PMC4003774

[62] Schumann GG (2007) APOBEC3 proteins: major players in intracellular defence against LINE-1-mediated retrotransposition. Biochem Soc Trans 35: 637–642. 17511669 10.1042/BST0350637

[63] Stetson DB, Ko JS, Heidmann T, Medzhitov R (2008) Trex1 prevents cell-intrinsic initiation of autoimmunity. Cell 134: 587–598. 10.1016/j.cell.2008.06.032 18724932 PMC2626626

[64] Arjan-Odedra S, Swanson CM, Sherer NM, Wolinsky SM, Malim MH (2012) Endogenous MOV10 inhibits the retrotransposition of endogenous retroelements but not the replication of exogenous retroviruses. Retrovirology 9: 53 10.1186/1742-4690-9-53 22727223 PMC3408377

[65] Goodier JL, Cheung LE, Kazazian HH Jr. (2012) MOV10 RNA helicase is a potent inhibitor of retrotransposition in cells. PLoS Genet 8: e1002941 10.1371/journal.pgen.1002941 23093941 PMC3475670

[66] Li X, Zhang J, Jia R, Cheng V, Xu X, et al (2013) The MOV10 helicase inhibits LINE-1 mobility. J Biol Chem 288: 21148–21160. 10.1074/jbc.M113.465856 23754279 PMC3774381

[67] Peddigari S, Li PW-L, Rabe JL, Martin SL (2013) hnRNPL and nucleolin bind LINE-1 RNA and function as host factors to modulate retrotransposition. Nucleic Acids Research 41: 575–585. 10.1093/nar/gks1075 23161687 PMC3592465

[68] Zhao K, Du J, Han X, Goodier JL, Li P, et al (2013) Modulation of LINE-1 and Alu/SVA retrotransposition by Aicardi-Goutieres syndrome-related SAMHD1. Cell Rep 4: 1108–1115. 10.1016/j.celrep.2013.08.019 24035396 PMC3988314

[69] Zhang A, Dong B, Doucet AJ, Moldovan JB, Moran JV, et al (2014) RNase L restricts the mobility of engineered retrotransposons in cultured human cells. Nucleic Acids Res 42: 3803–3820. 10.1093/nar/gkt1308 24371271 PMC3973342

[70] deHaro D, Kines KJ, Sokolowski M, Dauchy RT, Streva VA, et al (2014) Regulation of L1 expression and retrotransposition by melatonin and its receptor: implications for cancer risk associated with light exposure at night. Nucleic Acids Research 42: 7694–7707. 10.1093/nar/gku503 24914052 PMC4081101

[71] Wei W, Morrish TA, Alisch RS, Moran JV (2000) A transient assay reveals that cultured human cells can accommodate multiple LINE-1 retrotransposition events. Anal Biochem 284: 435–438. 10964437 10.1006/abio.2000.4675

[72] Gao G, Guo X, Goff SP (2002) Inhibition of retroviral RNA production by ZAP, a CCCH-type zinc finger protein. Science 297: 1703–1706. 12215647 10.1126/science.1074276

[73] Burdick R, Smith JL, Chaipan C, Friew Y, Chen J, et al (2010) P body-associated protein Mov10 inhibits HIV-1 replication at multiple stages. J Virol 84: 10241–10253. 10.1128/JVI.00585-10 20668078 PMC2937795

[74] Leeds P, Peltz SW, Jacobson A, Culbertson MR (1991) The product of the yeast UPF1 gene is required for rapid turnover of mRNAs containing a premature translational termination codon. Genes Dev 5: 2303–2314. 1748286 10.1101/gad.5.12a.2303

[75] Hui J, Stangl K, Lane WS, Bindereif A (2003) HnRNP L stimulates splicing of the eNOS gene by binding to variable-length CA repeats. Nat Struct Biol 10: 33–37. 12447348 10.1038/nsb875

[76] Kernan MJ, Kuroda MI, Kreber R, Baker BS, Ganetzky B (1991) napts, a mutation affecting sodium channel activity in Drosophila, is an allele of mle, a regulator of X chromosome transcription. Cell 66: 949–959. 1653649 10.1016/0092-8674(91)90440-a

[77] Kuroda MI, Kernan MJ, Kreber R, Ganetzky B, Baker BS (1991) The maleless protein associates with the X chromosome to regulate dosage compensation in Drosophila. Cell 66: 935–947. 1653648 10.1016/0092-8674(91)90439-6

[78] Bergemann AD, Ma ZW, Johnson EM (1992) Sequence of cDNA comprising the human pur gene and sequence-specific single-stranded-DNA-binding properties of the encoded protein. Mol Cell Biol 12: 5673–5682. 1448097 10.1128/mcb.12.12.5673-5682.1992 PMC360507

[79] Grana X, De Luca A, Sang N, Fu Y, Claudio PP, et al (1994) PITALRE, a nuclear CDC2-related protein kinase that phosphorylates the retinoblastoma protein in vitro. Proc Natl Acad Sci U S A 91: 3834–3838. 8170997 10.1073/pnas.91.9.3834 PMC43676

[80] Kao PN, Chen L, Brock G, Ng J, Kenny J, et al (1994) Cloning and expression of cyclosporin A- and FK506-sensitive nuclear factor of activated T-cells: NF45 and NF90. J Biol Chem 269: 20691–20699. 7519613

[81] Ashburner M, Ball CA, Blake JA, Botstein D, Butler H, et al (2000) Gene ontology: tool for the unification of biology. The Gene Ontology Consortium. Nat Genet 25: 25–29. 10802651 10.1038/75556 PMC3037419

[82] Castello A, Fischer B, Eichelbaum K, Horos R, Beckmann BM, et al (2012) Insights into RNA biology from an atlas of mammalian mRNA-binding proteins. Cell 149: 1393–1406. 10.1016/j.cell.2012.04.031 22658674

[83] Baltz AG, Munschauer M, Schwanhausser B, Vasile A, Murakawa Y, et al (2012) The mRNA-bound proteome and its global occupancy profile on protein-coding transcripts. Mol Cell 46: 674–690. 10.1016/j.molcel.2012.05.021 22681889

[84] Mandal PK, Ewing AD, Hancks DC, Kazazian HH Jr. (2013) Enrichment of processed pseudogene transcripts in L1-ribonucleoprotein particles. Hum Mol Genet 22: 3730–3748. 10.1093/hmg/ddt225 23696454 PMC3749862

[85] Kopera HC, Moldovan JB, Morrish TA, Garcia-Perez JL, Moran JV (2011) Similarities between long interspersed element-1 (LINE-1) reverse transcriptase and telomerase. Proc Natl Acad Sci U S A. 10.1073/pnas.1100275108 PMC3251045 21940498

[86] Muckenfuss H, Hamdorf M, Held U, Perkovic M, Lower J, et al (2006) APOBEC3 proteins inhibit human LINE-1 retrotransposition. J Biol Chem 281: 22161–22172. 16735504 10.1074/jbc.M601716200

[87] Bogerd HP, Wiegand HL, Hulme AE, Garcia-Perez JL, O'Shea KS, et al (2006) Cellular inhibitors of long interspersed element 1 and Alu retrotransposition. Proc Natl Acad Sci U S A 103: 8780–8785. 16728505 10.1073/pnas.0603313103 PMC1482655

[88] Chen H, Lilley CE, Yu Q, Lee DV, Chou J, et al (2006) APOBEC3A is a potent inhibitor of adeno-associated virus and retrotransposons. Curr Biol 16: 480–485. 16527742 10.1016/j.cub.2006.01.031

[89] Goodier JL, Cheung LE, Kazazian HH Jr. (2013) Mapping the LINE1 ORF1 protein interactome reveals associated inhibitors of human retrotransposition. Nucleic Acids Res 41: 7401–19. 10.1093/nar/gkt512 23749060 PMC3753637

[90] Kerns JA, Emerman M, Malik HS (2008) Positive selection and increased antiviral activity associated with the PARP-containing isoform of human zinc-finger antiviral protein. PLoS Genet 4: e21 10.1371/journal.pgen.0040021 18225958 PMC2213710

[91] Chen S, Xu Y, Zhang K, Wang X, Sun J, et al (2012) Structure of N-terminal domain of ZAP indicates how a zinc-finger protein recognizes complex RNA. Nat Struct Mol Biol 19: 430–435. 10.1038/nsmb.2243 22407013

[92] Guo X, Carroll J-WN, MacDonald MR, Goff SP, Gao G (2004) The Zinc Finger Antiviral Protein Directly Binds to Specific Viral mRNAs through the CCCH Zinc Finger Motifs. Journal of Virology 78: 12781–12787. 15542630 10.1128/JVI.78.23.12781-12787.2004 PMC525010

[93] Ullu E, Tschudi C (1984) Alu sequences are processed 7SL RNA genes. Nature 312: 171–172. 6209580 10.1038/312171a0

[94] Goodier JL, Ostertag EM, Du K, Kazazian HH Jr. (2001) A novel active L1 retrotransposon subfamily in the mouse. Genome Res 11: 1677–1685. 11591644 10.1101/gr.198301 PMC311137

[95] Sugano T, Kajikawa M, Okada N (2006) Isolation and characterization of retrotransposition-competent LINEs from zebrafish. Gene 365: 74–82. 16356661 10.1016/j.gene.2005.09.037

[96] Han JS, Boeke JD (2004) A highly active synthetic mammalian retrotransposon. Nature 429: 314–318. 15152256 10.1038/nature02535

[97] Ostertag EM, Prak ET, DeBerardinis RJ, Moran JV, Kazazian HH Jr. (2000) Determination of L1 retrotransposition kinetics in cultured cells. Nucleic Acids Res 28: 1418–1423. 10684937 10.1093/nar/28.6.1418 PMC111040

[98] Martin SL, Cruceanu M, Branciforte D, Wai-Lun Li P, Kwok SC, et al (2005) LINE-1 retrotransposition requires the nucleic acid chaperone activity of the ORF1 protein. J Mol Biol 348: 549–561. 15826653 10.1016/j.jmb.2005.03.003

[99] Belancio VP, Roy-Engel AM, Deininger P (2008) The impact of multiple splice sites in human L1 elements. Gene 411: 38–45. 10.1016/j.gene.2007.12.022 18261861 PMC2278003

[100] Belancio VP, Hedges DJ, Deininger P (2006) LINE-1 RNA splicing and influences on mammalian gene expression. Nucleic Acids Research 34: 1512–1521. 16554555 10.1093/nar/gkl027 PMC1415225

[101] Perepelitsa-Belancio V, Deininger P (2003) RNA truncation by premature polyadenylation attenuates human mobile element activity. Nat Genet 35: 363–366. 14625551 10.1038/ng1269

[102] Hohjoh H, Singer MF (1997) Ribonuclease and high salt sensitivity of the ribonucleoprotein complex formed by the human LINE-1 retrotransposon. J Mol Biol 271: 7–12. 9300051 10.1006/jmbi.1997.1159

[103] Wissing S, Montano M, Garcia-Perez JL, Moran JV, Greene WC (2011) Endogenous APOBEC3B Restricts LINE-1 Retrotransposition in Transformed Cells and Human Embryonic Stem Cells. Journal of Biological Chemistry 286: 36427–36437. 10.1074/jbc.M111.251058 21878639 PMC3196128

[104] Goodier JL, Zhang L, Vetter MR, Kazazian HH Jr. (2007) LINE-1 ORF1 protein localizes in stress granules with other RNA-binding proteins, including components of RNA interference RNA-induced silencing complex. Mol Cell Biol 27: 6469–6483. 17562864 10.1128/MCB.00332-07 PMC2099616

[105] Liu L, Chen G, Ji X, Gao G (2004) ZAP is a CRM1-dependent nucleocytoplasmic shuttling protein. Biochemical and biophysical research communications 321: 517–523. 15358138 10.1016/j.bbrc.2004.06.174

[106] Garcia-Perez JL, Morell M, Scheys JO, Kulpa DA, Morell S, et al (2010) Epigenetic silencing of engineered L1 retrotransposition events in human embryonic carcinoma cells. Nature 466: 769–773. 10.1038/nature09209 20686575 PMC3034402

[107] Zeuthen J, Norgaard JO, Avner P, Fellous M, Wartiovaara J, et al (1980) Characterization of a human ovarian teratocarcinoma-derived cell line. Int J Cancer 25: 19–32. 6931103 10.1002/ijc.2910250104

[108] Leung AK, Vyas S, Rood JE, Bhutkar A, Sharp PA, et al (2011) Poly(ADP-ribose) regulates stress responses and microRNA activity in the cytoplasm. Mol Cell 42: 489–499. 10.1016/j.molcel.2011.04.015 21596313 PMC3898460

[109] Guo X, Ma J, Sun J, Gao G (2007) The zinc-finger antiviral protein recruits the RNA processing exosome to degrade the target mRNA. Proc Natl Acad Sci U S A 104: 151–156. 17185417 10.1073/pnas.0607063104 PMC1765426

[110] Belancio VP, Roy-Engel AM, Pochampally RR, Deininger P (2010) Somatic expression of LINE-1 elements in human tissues. Nucleic Acids Res 38: 3909–3922. 10.1093/nar/gkq132 20215437 PMC2896524

[111] Zhu Y, Chen G, Lv F, Wang X, Ji X, et al (2011) Zinc-finger antiviral protein inhibits HIV-1 infection by selectively targeting multiply spliced viral mRNAs for degradation. Proc Natl Acad Sci U S A 108: 15834–15839. 10.1073/pnas.1101676108 21876179 PMC3179061

[112] Bick MJ, Carroll JW, Gao G, Goff SP, Rice CM, et al (2003) Expression of the zinc-finger antiviral protein inhibits alphavirus replication. J Virol 77: 11555–11562. 14557641 10.1128/JVI.77.21.11555-11562.2003 PMC229374

[113] Zhu Y, Wang X, Goff SP, Gao G (2012) Translational repression precedes and is required for ZAP-mediated mRNA decay. EMBO J 31: 4236–4246. 10.1038/emboj.2012.271 23023399 PMC3492732

[114] Chen G, Guo X, Lv F, Xu Y, Gao G (2008) p72 DEAD box RNA helicase is required for optimal function of the zinc-finger antiviral protein. Proc Natl Acad Sci U S A 105: 4352–4357. 10.1073/pnas.0712276105 18334637 PMC2393818

[115] Buchan JR, Parker R (2009) Eukaryotic Stress Granules: The Ins and Outs of Translation. Molecular Cell 36: 932–941. 10.1016/j.molcel.2009.11.020 20064460 PMC2813218

[116] Reineke LC, Lloyd RE (2013) Diversion of stress granules and P-bodies during viral infection. Virology 436: 255–267. 10.1016/j.virol.2012.11.017 23290869 PMC3611887

[117] Bick MJ, Carroll J-WN, Gao G, Goff SP, Rice CM, et al (2003) Expression of the Zinc-Finger Antiviral Protein Inhibits Alphavirus Replication. Journal of Virology 77: 11555–11562. 14557641 10.1128/JVI.77.21.11555-11562.2003 PMC229374

[118] Muller S, Moller P, Bick MJ, Wurr S, Becker S, et al (2007) Inhibition of filovirus replication by the zinc finger antiviral protein. J Virol 81: 2391–2400. 17182693 10.1128/JVI.01601-06 PMC1865956

[119] Mao R, Nie H, Cai D, Zhang J, Liu H, et al (2013) Inhibition of hepatitis B virus replication by the host zinc finger antiviral protein. PLoS Pathog 9: e1003494 10.1371/journal.ppat.1003494 23853601 PMC3708887

[120] Burton FH, Loeb DD, Voliva CF, Martin SL, Edgell MH, et al (1986) Conservation throughout mammalia and extensive protein-encoding capacity of the highly repeated DNA long interspersed sequence one. J Mol Biol 187: 291–304. 3009828 10.1016/0022-2836(86)90235-4

[121] Smit AF, Toth G, Riggs AD, Jurka J (1995) Ancestral, mammalian-wide subfamilies of LINE-1 repetitive sequences. J Mol Biol 246: 401–417. 7877164 10.1006/jmbi.1994.0095

[122] Yang L, Brunsfeld J, Scott L, Wichman H (2014) Reviving the dead: history and reactivation of an extinct l1. PLoS Genet 10: e1004395 10.1371/journal.pgen.1004395 24968166 PMC4072516

[123] Sawyer SL, Emerman M, Malik HS (2004) Ancient adaptive evolution of the primate antiviral DNA-editing enzyme APOBEC3G. PLoS Biol 2: E275 15269786 10.1371/journal.pbio.0020275 PMC479043

[124] Sawyer SL, Wu LI, Emerman M, Malik HS (2005) Positive selection of primate TRIM5alpha identifies a critical species-specific retroviral restriction domain. Proc Natl Acad Sci U S A 102: 2832–2837. 15689398 10.1073/pnas.0409853102 PMC549489

[125] Sawyer SL, Malik HS (2006) Positive selection of yeast nonhomologous end-joining genes and a retrotransposon conflict hypothesis. Proc Natl Acad Sci U S A 103: 17614–17619. 17101967 10.1073/pnas.0605468103 PMC1693795

[126] Hulme AE, Bogerd HP, Cullen BR, Moran JV (2007) Selective inhibition of Alu retrotransposition by APOBEC3G. Gene 390: 199–205. 17079095 10.1016/j.gene.2006.08.032 PMC2917221

[127] Dombroski BA, Scott AF, Kazazian HH Jr. (1993) Two additional potential retrotransposons isolated from a human L1 subfamily that contains an active retrotransposable element. Proc Natl Acad Sci U S A 90: 6513–6517. 8393568 10.1073/pnas.90.14.6513 PMC46962

[128] Freeman JD, Goodchild NL, Mager DL (1994) A modified indicator gene for selection of retrotransposition events in mammalian cells. Biotechniques 17: 46, 48–49, 52 7946311

[129] Wallace MR, Andersen LB, Saulino AM, Gregory PE, Glover TW, et al (1991) A de novo Alu insertion results in neurofibromatosis type 1. Nature 353: 864–866. 1719426 10.1038/353864a0

[130] Esnault C, Casella JF, Heidmann T (2002) A Tetrahymena thermophila ribozyme-based indicator gene to detect transposition of marked retroelements in mammalian cells. Nucleic Acids Res 30: e49 12034850 10.1093/nar/30.11.e49 PMC117211

[131] Bogerd HP, Wiegand HL, Doehle BP, Lueders KK, Cullen BR (2006) APOBEC3A and APOBEC3B are potent inhibitors of LTR-retrotransposon function in human cells. Nucleic Acids Res 34: 89–95. 16407327 10.1093/nar/gkj416 PMC1326241

[132] Liu J, Valencia-Sanchez MA, Hannon GJ, Parker R (2005) MicroRNA-dependent localization of targeted mRNAs to mammalian P-bodies. Nat Cell Biol 7: 719–723. 15937477 10.1038/ncb1274 PMC1855297

[133] Tourriere H, Chebli K, Zekri L, Courselaud B, Blanchard JM, et al (2003) The RasGAP-associated endoribonuclease G3BP assembles stress granules. J Cell Biol 160: 823–831. 12642610 10.1083/jcb.200212128 PMC2173781

[134] Shevchenko A, Wilm M, Vorm O, Mann M (1996) Mass Spectrometric Sequencing of Proteins from Silver-Stained Polyacrylamide Gels. Analytical Chemistry 68: 850–858. 8779443 10.1021/ac950914h

[135] Licklider LJ, Thoreen CC, Peng J, Gygi SP (2002) Automation of Nanoscale Microcapillary Liquid Chromatography,àíTandem Mass Spectrometry with a Vented Column. Analytical Chemistry 74: 3076–3083. 12141667 10.1021/ac025529o

[136] Rauch A, Bellew M, Eng J, Fitzgibbon M, Holzman T, et al (2005) Computational Proteomics Analysis System (CPAS): An Extensible, Open-Source Analytic System for Evaluating and Publishing Proteomic Data and High Throughput Biological Experiments. Journal of Proteome Research 5: 112–121. 10.1021/pr0503533 16396501

[137] Craig R, Beavis RC (2004) TANDEM: matching proteins with tandem mass spectra. Bioinformatics 20: 1466–1467. 14976030 10.1093/bioinformatics/bth092

[138] Nesvizhskii AI, Keller A, Kolker E, Aebersold R (2003) A Statistical Model for Identifying Proteins by Tandem Mass Spectrometry. Analytical Chemistry 75: 4646–4658. 14632076 10.1021/ac0341261

[139] Keller A, Nesvizhskii AI, Kolker E, Aebersold R (2002) Empirical Statistical Model To Estimate the Accuracy of Peptide Identifications Made by MS/MS and Database Search. Analytical Chemistry 74: 5383–5392. 12403597 10.1021/ac025747h

[140] Schneider CA, Rasband WS, Eliceiri KW (2012) NIH Image to ImageJ: 25 years of image analysis. Nat Methods 9: 671–675. 22930834 10.1038/nmeth.2089 PMC5554542

