# Supplementary figures and images for: The Zinc-Finger Antiviral Protein ZAP Inhibits LINE and Alu Retrotransposition
Source: PLoS Genet. 2015 May 7;11(5):e1005121. doi: 10.1371/journal.pgen.1005121 (PMC4423928; doi:10.1371/journal.pgen.1005121)

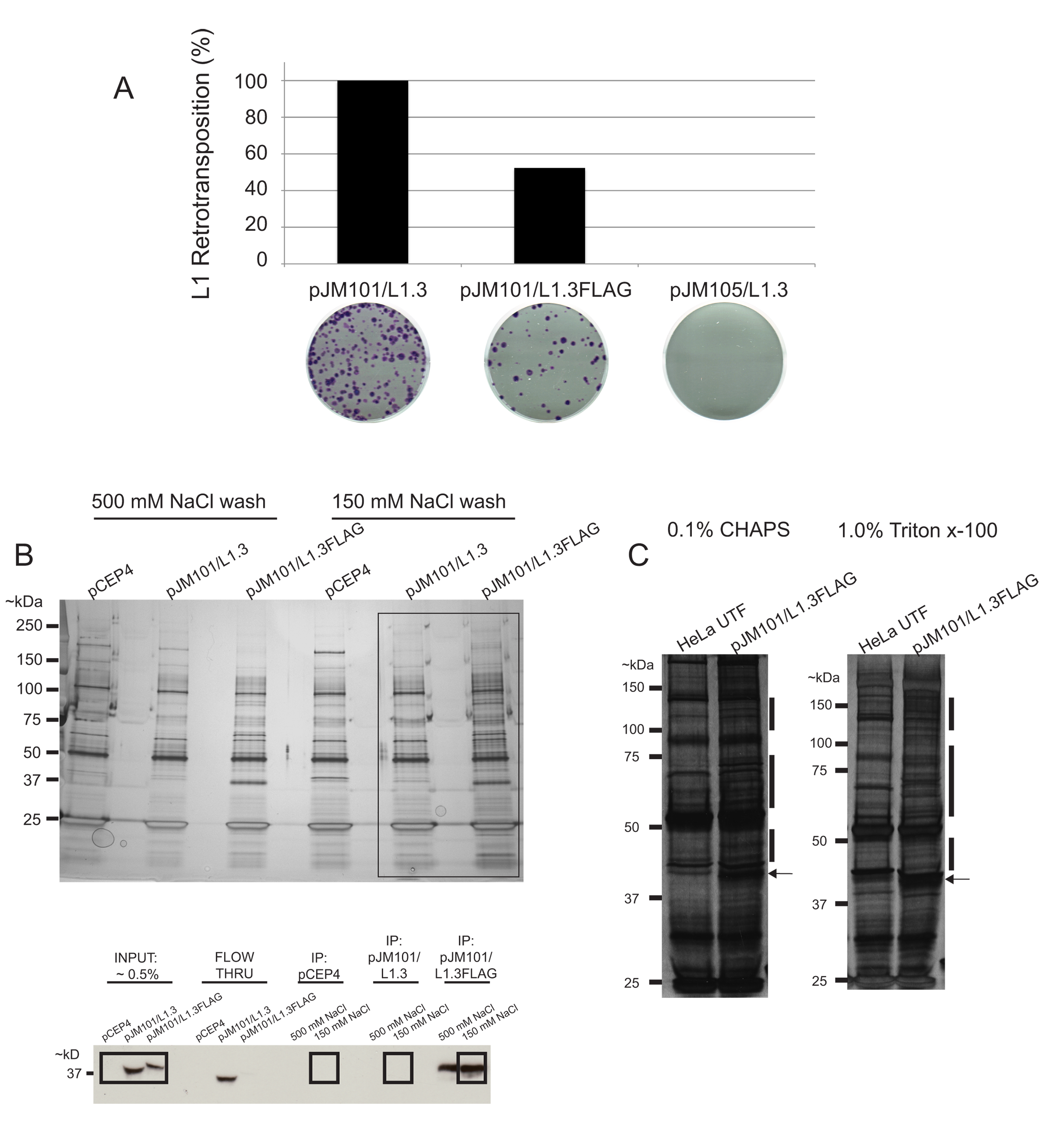

Supplement: S1 Fig — (A) The FLAG epitope on ORF1p is compatible with retrotransposition: Constructs were tested in a transient HeLa cell retrotransposition assay [30,71]. The X-axis indicates the L1 plasmid. The Y-axis indicates the retrotransposition efficiency. Retrotransposition assays were normalized to pJM101/L1.3 (100%). The pJM105/L1.3 plasmid serves as a negative control and harbors a point mutation in the ORF2p RT domain that renders the element inactive [30]. Representative results from a single experiment are depicted below the graph. The assay was repeated two times with similar results. (B) Immunoprecipitation reactions conducted using various wash conditions: Top panel: HeLa cells were transfected with pCEP4, pJM101/L1.3, or pJM101/L1.3FLAG and were subjected to lysis using two different salt concentrations (500 mM NaCl (left gel) or 150 mM NaCl (right gel)). Shown are the images of silver stained gels from immunoprecipitation reactions. The black rectangles indicate the cropped image depicted in Fig 1B. Molecular weight standards (~kDa) are shown on the left side of the gel. Bottom panel: Image of full western blot used in Fig 1C. The black rectangle indicates the cropped lanes depicted in Fig 1C. Molecular weight standards (~kDa) are shown on the left side of the gel. (C) Immunoprecipitation reactions under different lysis buffer conditions: Silver stained gels of IP fractions from untransfected HeLa (HeLa UTF) or HeLa cells transfected with pJM101/L1.3FLAG. Lysis buffer contained either 0.1% CHAPS (left gel) or 1.0% Triton X-100 (right gel). Black arrows correspond to the approximate location of ORF1p-FLAG; black bars indicate the approximate location of proteins enriched in the pJM101/L1.3FLAG lane. Molecular weight standards (kDa) are shown on the left side of the gels. (TIF) [file pgen.1005121.s001.tif]

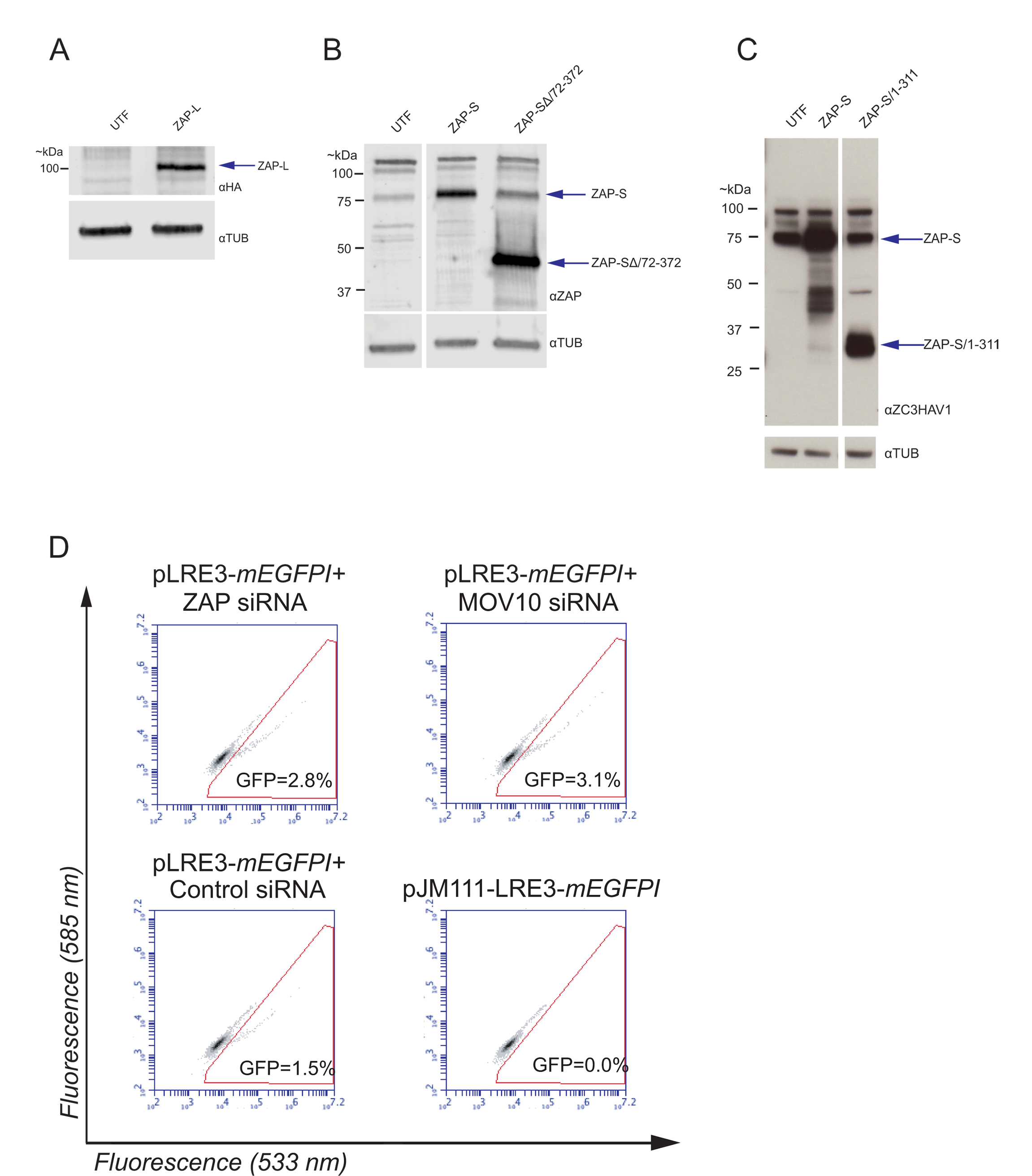

Supplement: S2 Fig — (A-C) Transfected ZAP is expressed in HeLa cells: Western blots of whole cell lysates demonstrate the expression of ZAP-L (panel A) and ZAP-S and ZAP-S/∆72–372 (panels B and C) 48 hours post-transfection. UTF indicates untransfected HeLa cells. The antibodies are indicated at the right side of the blots. Blue arrows indicate the approximate locations of the ZAP proteins. Tubulin serves as a loading control. Molecular weight standards (kDa) are shown on the left side of the blots. (D) The depletion of endogenous ZAP enhances L1 retrotransposition: Flow cytometry was used to determine the percentage of EGFP-positive, live-gated cells for each siRNA transfection condition (noted above the plots). The X-axis depicts the scattering at 533 nm; the Y-axis depicts the scattering at 585 nm. The EGFP-positive gate was set using the retrotransposition-deficient negative control, pJM111-LRE3-mEGFPI [30,69]. (TIF) [file pgen.1005121.s002.tif]

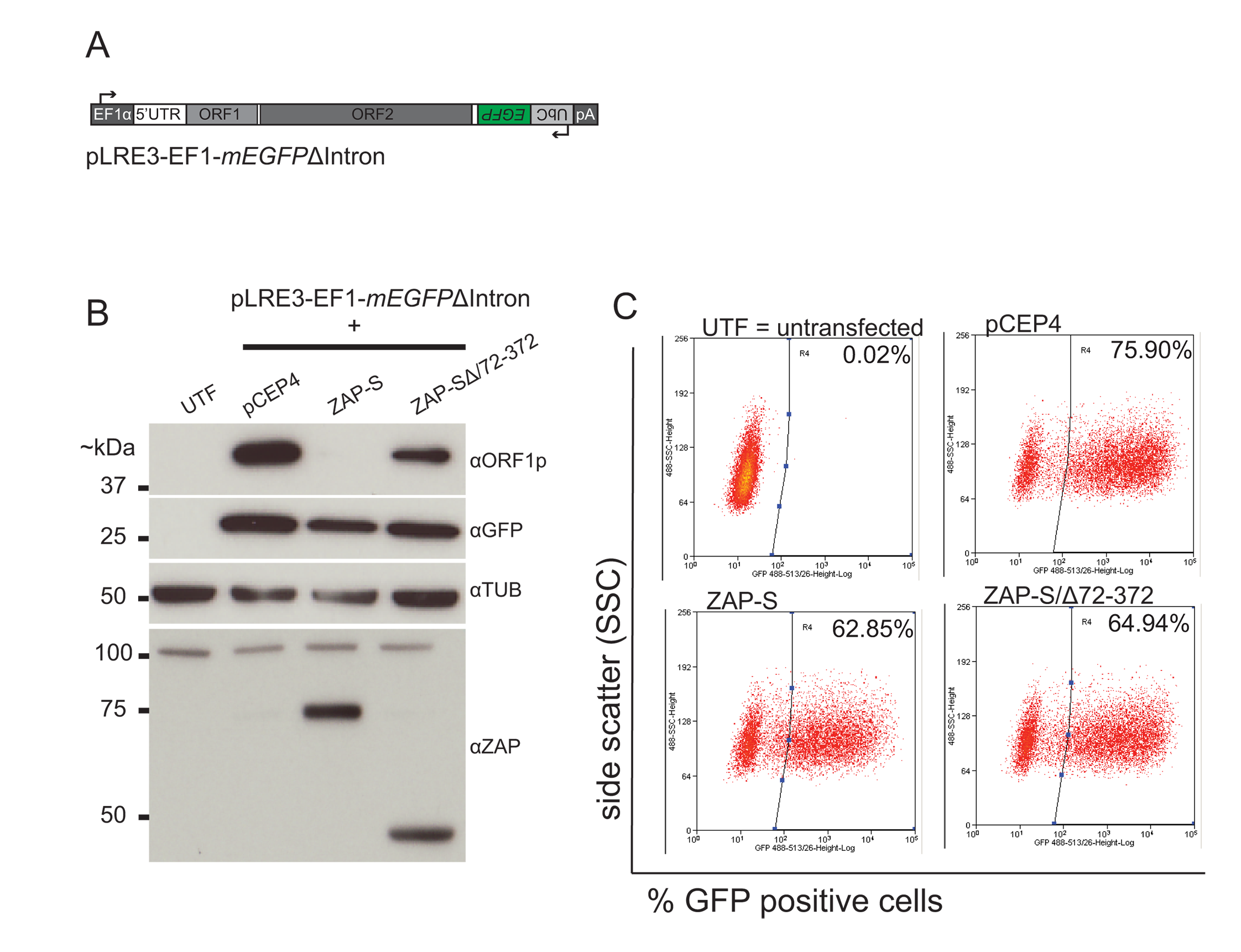

Supplement: S3 Fig — ZAP-S preferentially suppresses the expression of ORF1p. (A) A schematic of the pLRE3-EF1-mEGFPΔIntron: pLRE3-EF1-mEGFPΔIntron expresses a human L1 (LRE3) that is tagged with an mEGFPI expression cassette that lacks an intron. The human elongation factor-1 alpha (EF1α) promoter (arrow) augments L1 transcription. The ubiquitin C (UbC) promoter (upside down arrow) drives EGFP transcription. (B) ZAP-S inhibits ORF1p expression: Western blots were conducted using whole cell lysates derived from cells co-transfected with pLRE3-EF1-mEGFPΔIntron and the ZAP-S expression plasmid or pCEP4 indicated above each lane. UTF indicates whole cell lysates from untransfected HeLa cell. Antibodies are indicated on the right side of each blot. Tubulin is used as a loading control. Western blot images depict a representative experiment that was repeated three times with similar results. Notably, upon extended exposure times ORF1p was able to be visualized in the ZAP-S lane. (C) ZAP-S does not inhibit EFGP expression and/or accumulation: HeLa cells were co-transfected with pLRE3-EF1-mEGFPΔIntron and the indicated expression plasmids (noted above the plots). Flow cytometry was used to determine the percentage of EGFP-positive, live-gated cells for each condition. UTF indicates untransfected HeLa cells. The EGFP-positive gate was set using the UTF sample as a negative control. The X-axis depicts the percentage of EGFP positive cells. The Y-axis indicates the side scattering profile (SSC). Approximately 1.2–1.7 x 10^6^ GFP positive cells were collected and analyzed for each transfection condition. (TIF) [file pgen.1005121.s003.tif]

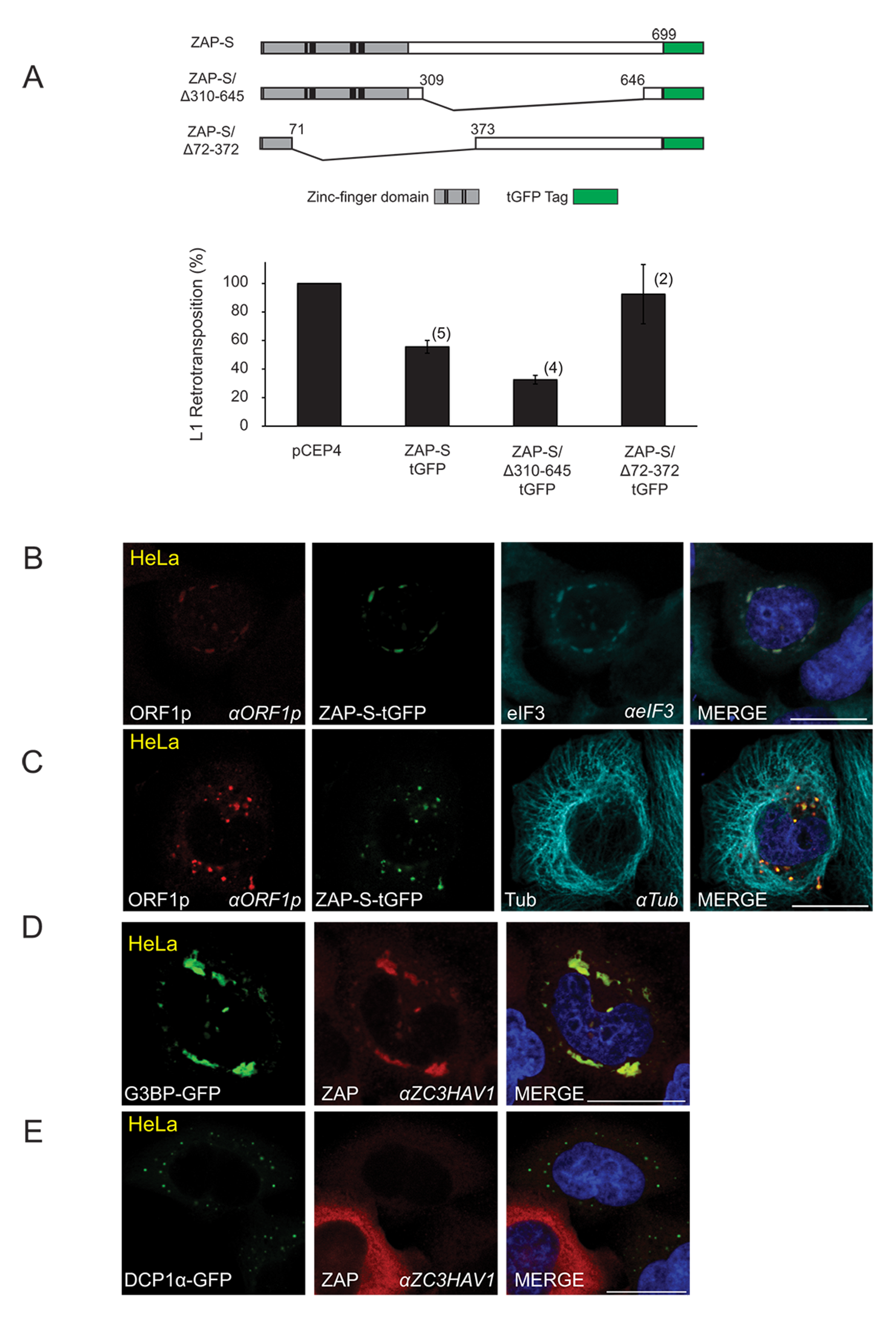

Supplement: S4 Fig — (A) ZAP-S-tGFP inhibits retrotransposition in HeLa cells: Top panel: Schematics of tGFP-tagged ZAP constructs. Depicted are the relative positions of the zinc-finger domains (light gray rectangles), cysteine-histidine (CCCH) zinc-fingers (vertical black bars), and tGFP tag (green rectangles) ZAP-S expression constructs. Bottom panel: Results of pJJ101/L1.3 retrotransposition assays. The X-axis indicates the cDNA co-transfected with pJJ101/L1.3 or pcDNA6/TR. The Y-axis indicates pJJ101/L1.3 retrotransposition activity (black bars). All values have been normalized to the pCEP4 empty vector control (100%). The numbers above the bar graphs indicate the number of biological replicates performed with each cDNA expression construct. Error bars represent standard deviations. (B-E) ORF1p and ZAP co-localize with stress granules in HeLa cells: HeLa cells were co-transfected with pJM101/L1.3Δneo and ZAP-S-tGFP; proteins were visualized by direct immunofluorescence. ORF1p co-localizes with ectopic ZAP-S-tGFP and eIF3 in cytoplasmic foci (panel B). ORF1p co-localizes with ectopic ZAP-S-tGFP (panels A and B), but not with tubulin (panel C). GFP-tagged G3BP co-localizes with endogenous ZAP in cytoplasmic foci (panel D). GFP-tagged DCP1α forms cytoplasmic punctate structures, which do not appear to co-localize with endogenous ZAP (panel E). The right-most image in each panel represents a merged image. The cell type is indicated at the top left (yellow), the protein name is listed on the bottom left, and the name of the primary antibody used (italicized) is annotated at the bottom right. Nuclei were stained with DAPI (blue) and the scale bar represents 25 μM. (TIF) [file pgen.1005121.s004.tif]

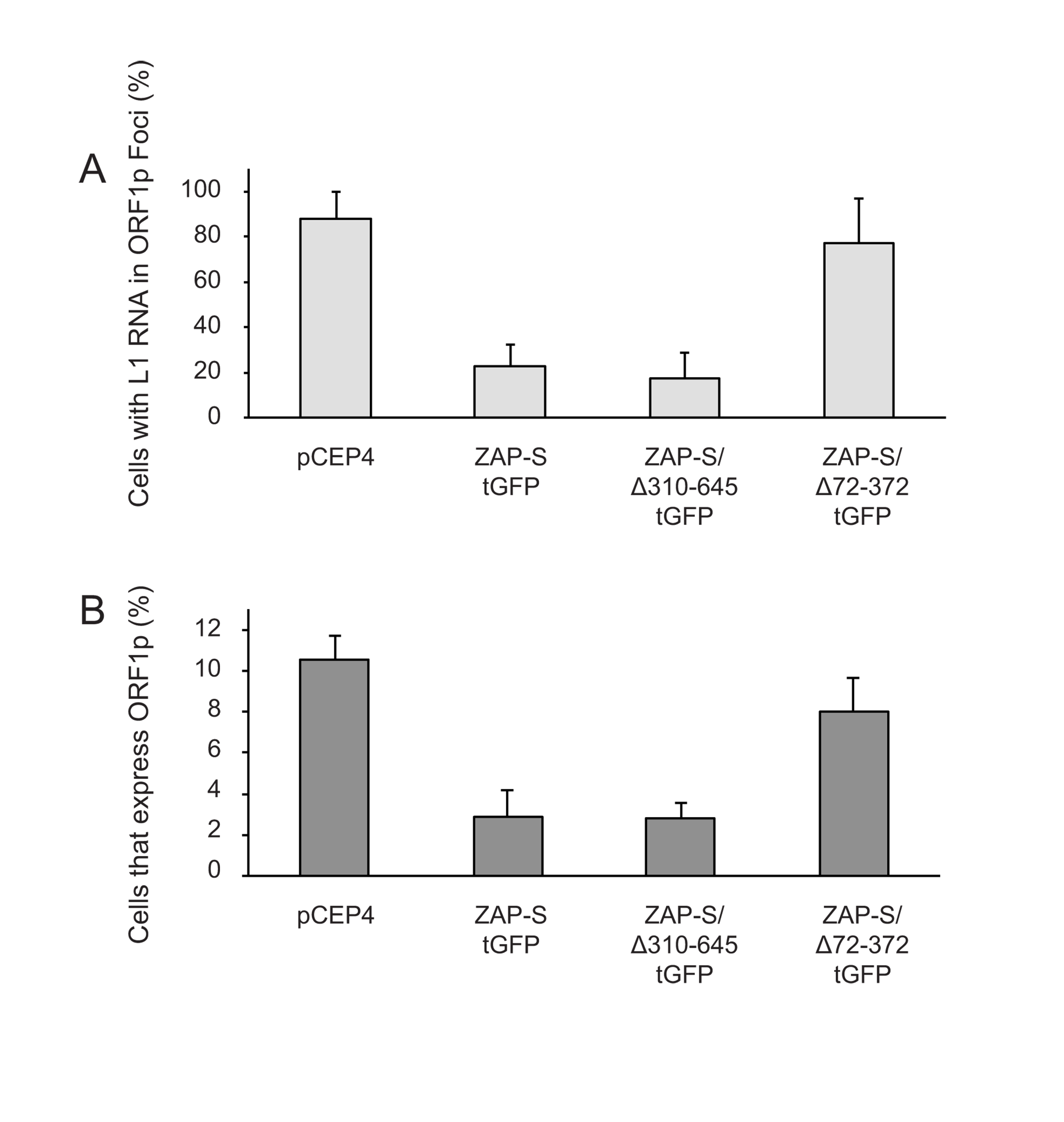

Supplement: S5 Fig — (A) Fluorescence microscopy was used to determine the percentage of cells that contained L1 RNA in cytoplasmic ORF1p foci. The X-axis indicates the plasmid that was co-transfected with pJM101/L1.3. The Y-axis of the graph depicts the percentage of cells where L1 RNA was detected in cytoplasmic ORF1p foci. Experiments were repeated three times. A total of ~60 visual fields (~1600 cells) were examined amongst all three experiments and ~33–41 ORF1p foci containing cells were evaluated for each experimental condition. Error bars represent standard deviations. (B) Confocal microscopy was used to determine the number of ORF1p-expressing cells ~48 hours post transfection. The X-axis indicates the plasmid that was co-transfected with pJM101/L1.3Δneo. The Y-axis of the graph depicts the percentage of cells that express ORF1p. Experiments were repeated twice. Each experiment contained two biological replicates and ~1100–1500 cells were enumerated amongst all experiments for each condition. Error bars indicate standard deviations. (TIF) [file pgen.1005121.s005.tif]

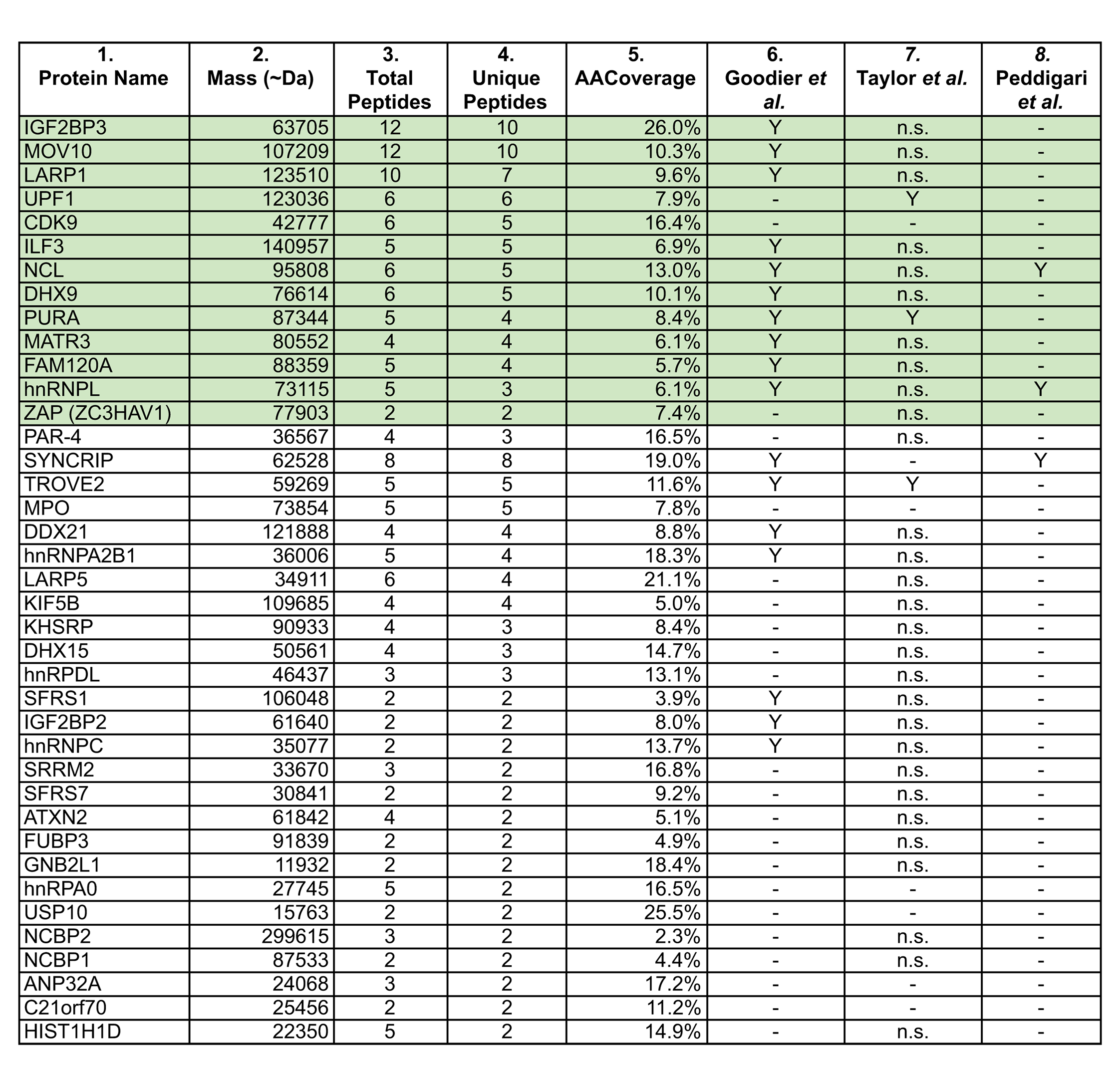

Supplement: S1 Table — ORF1p-interacting proteins were selected based on the criteria that the protein was unique to the pJM101/L1.3FLAG IP and was identified by two or more unique peptides (peptide error ≤0.05; protein probability ≥0.95). Column 1 = protein name. Column 2 = protein mass. Column 3 = total number of identified peptides. Column 4 = number of unique peptides. Column 5 = the percentage of amino acid coverage for each of the respective proteins. Columns 6–8 = whether the proteins were identified in the indicated studies [47,67,89]. Green highlighting indicates ORF1p-interacting candidates that were verified by western blot (Fig 1D). "Y" in columns 6–8 indicate that the protein was identified as a significant L1-interacting protein by statistical and/or direct biochemical methods; “n.s.” in column 7 indicates that the protein was identified in the study, but that it did not reach the significance threshold set by the authors. (TIF) [file pgen.1005121.s006.tif]
